# Analysis of Fentanyl and Fentanyl Analogs Using Atmospheric Pressure Chemical Ionization Gas Chromatography–Mass Spectrometry (APCI-GC-MS)

**DOI:** 10.1021/jasms.4c00455

**Published:** 2025-02-03

**Authors:** Karen A. Reyes Monroy, Richard McCrary, Isabelle Parry, Catherine Webber, Teresa D. Golden, Guido F. Verbeck

**Affiliations:** Department of Chemistry, University of North Texas, 1155 Union Circle #30570, Denton, Texas 76203, United States

**Keywords:** Atmospheric Pressure Chemical Ionization, Mass Spectrometry, Gas Chromatography, Fentanyl, Analogs, Fragmentation Analysis

## Abstract

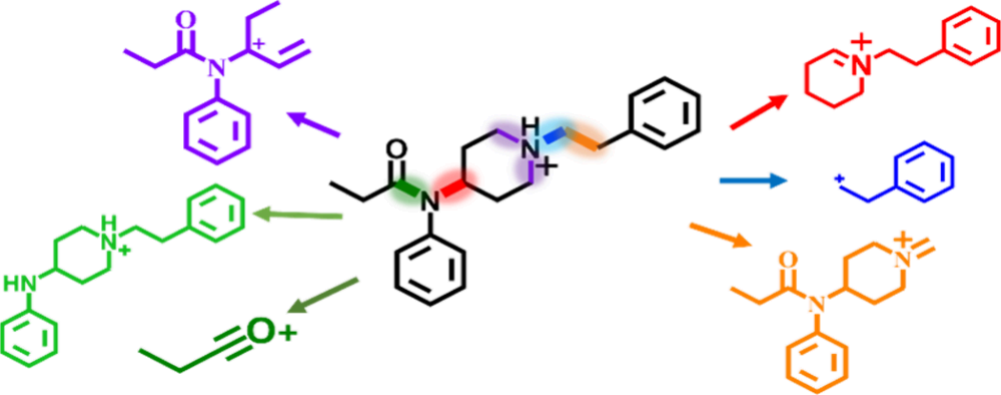

Illicit fentanyl and fentanyl analogs are a growing concern in the United States as opioid related deaths rise. Given that fentanyl analogs are readily obtained by modifying the structure of fentanyl, illicit fentanyl analogs appearing on the black market often contain similar structures, making analogue differentiation and identification difficult. Thus, obtaining both precursor and product ion data during analysis is becoming increasingly valuable in fentanyl analog characterization. In this paper, we provide GC column retention time, precursor, and product ion mass spectrum data for 74 fentanyl analogs that were analyzed using atmospheric pressure chemical ionization-gas chromatography–mass spectrometry (APCI-GC-MS) utilizing a triple quadrupole mass analyzer. During analysis, precursor ions underwent collision induced dissociation (CID) by increasing the collision energy (10, 20, 30, 40, and 50 V) throughout a single run. Data reveal that APCI readily produces product ions of the piperidine and *N*-alkyl chain but rarely provides data on the acyl group. Furthermore, fentanyl analogs with greater substitution at the *N*-alkyl chain demonstrate a greater preference for dissociation at the N-αC and αC-βC bond, while greater substitution at the amide group leads to fragmentation at the N–C4 bond.

## Introduction

Soon after its discovery, fentanyl, which is a synthetic opioid originally developed by Dr. Paul Janssen in 1960, was approved for use as pain relief medication and as an anesthetic in medical settings.^1,2^ Shortly after its integration into the medical field, fentanyl abuse as well as overdoses began to increase, and this caused stricter measures to be placed on the pharmaceutical use of fentanyl, which made it a scheduled II drug.^3^ The demand for this highly addictive and potent opioid continued to increase well after stricter measures were placed on its medicinal use.^3,4^ The Drug Enforcement Administration (DEA) began to see fentanyl arrive in the illicit drug market; this posed a great risk to the community, since, like morphine, fentanyl is a potent analgesic but is 50–100 times more powerful and serves as the foundation for a family of analogs.^5−9^ To mitigate the production of illicit fentanyl, the DEA placed restrictions on commonly used precursors of the drug such as 4-anilino-N-phenethylpiperidine (A-NPP).^5^ Data accumulated by the DEA show that fentanyl is rarely produced within the states and is commonly made in foreign laboratories. The majority of fentanyl-producing laboratories are in China; these laboratories can either directly export the fentanyl to the US or export it to Mexico for transit to the US.^10−12^ To evade current laws against fentanyl, clandestine laboratories are implementing new reagents and precursors to obtain fentanyl analogs. The core structure of fentanyl is readily modified; therefore, structural modifications of the base structure of fentanyl are quite unchallenging.^13,14^ Clandestine manufacturers implement chemical reactions using a wide variety of reagents to modify the core structure of fentanyl. Such practices produce analogs with different structures and unknown potencies, which is dangerous since fentanyl is increasingly used as a “cutting agent”.^15−17^ Fentanyl as a cutting agent poses a significant threat because of its high potency, making the chance of a drug overdose higher. In 2022, fentanyl and xylazine drug mixtures were found in multiple intoxication deaths; both of these drugs have narrow therapeutic windows and thus increase the chance of overdoses.^18^ While cutting agents are used primarily for economic reasons such as for maximizing drug profits, they pose a public health hazard because the users may not be aware of the substances they are ingesting or the true potency of the drugs they consume.^19^ This increases the likelihood of overdoses, adverse reactions, and fatalities.^5,20,21^

Fentanyl and its analogs act upon μ-opioid receptors and manifest significant psychoactive effects including profound sedation, loss of consciousness, and, in severe cases, death.^22,23^ The potency of these fentanyl analogs varies significantly; for example, carfentanil exhibits potency levels 5,000–10,000 times higher than those of morphine, while sufentanil is only 500 times more potent.^24^ According to the National Center for Health Statistics, in 2022, 22.7 deaths per 100,000 standard population from synthetic opioids other than methadone were reported in the US.^25^ Structural characterization of fentanyl analogs is crucial for determining its analgesic and anesthetic effects. Such information can be used to help treat fentanyl analogue overdoses with naloxone, an opioid antagonist that binds to opioid receptors, which blocks and reverses the effects of other opioids but has no effect on people who have no opioids in their system. Structural identification can be used to enact the “Anti-Drug Abuse Act of 1986″ which allows for the prosecution of structurally similar chemical compounds as schedule 1 or 2 controlled substances if it is intended for human consumption.^26^ Yet, as more new psychoactive substances (NPS) emerge, the ability to characterize them is becoming more strenuous; this in turn complicates the process of identifying the appropriate treatment and potential risks involved. There are many ways to identify fentanyl and its analogs. Characterization of these analogs often requires analysis of product ions produced, and for this reason, gas chromatography-electron ionization-mass spectrometry (GC-EI-MS) is currently the gold standard for illicit drug characterization.^27−30^ In GC, gas-phase molecules are introduced into the mass spectrometer under a high vacuum. These neutral molecules are then bombarded with electrons from a heated filament, causing them to ionize and fragment.^31−33^ EI is considered a hard ionization technique because it ionizes the molecule of interest directly, and this causes a lack of or low abundance of molecular ions produced.^34−40^ Thus, during GC-EI-MS analysis of fentanyl structures, the GC retention time and the product ions observed are used to determine the molecular ion, which is often absent in an EI spectrum. A major reason this technique is widely used is that resulting EI mass spectra can be standardized since each molecule has a unique “fingerprint”. Thus, drug identification using this technique can readily be accomplished if reference data is available.

Yet, while electron ionization generates fingerprint data that is vital during analogue identification, as previously stated, it produces little to no molecular ion data, which can simplify the characterization process. This presents a challenge when several fentanyl analogs are observed to undergo similar dissociation pathways. The lack of molecular ion mass-to-charge (*m*/*z*) peaks makes the identity assignment difficult. Furthermore, identification becomes more cumbersome when new and emerging drugs that do not have reference data are analyzed. Thus, while GC-EI-MS is a great tool for identifying known fentanyl substances, due to the lack of reference data for emerging substances, the same cannot be said for new and emerging drugs. Unlike EI, in gas chromatography chemical ionization (CI), a high abundance of intact molecular ion species is generated.^41−44^ Yet, this “soft” ionization technique can more easily ionize low polarity analytes that are not as readily ionized in ESI.^45^ Furthermore, its sensitivity is much lower than that of EI in GC-MS analysis due to reagent gas interference. The appeal for a soft ion source capable of producing an abundance of molecular ions as well as fragment data with high sensitivity and selectivity brought about the development of atmospheric pressure chemical ionization (APCI).

APCI is a powerful instrumental analysis technique known for its high sensitivity, high selectivity, high resolution, and soft ionization capabilities.^46−48^ It is typically used in conjunction with liquid chromatography–mass spectrometry (LC-MS), but it has also been coupled to GC-MS. Furthermore, APCI can generate both protonated and deprotonated molecular ions, as well as full dissociation data which is very beneficial for drug profiling and screening applications.^45,49−51^ The identity of analytes can thus be determined based on the molecular ion’s mass and/or the protonated molecule and produced fragments.^52^ This is extremely valuable for the analysis and characterization of new and emerging designer drugs that have never been cataloged.

**Figure 1:**
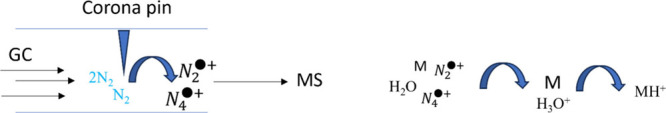
Atmospheric pressure chemical ionization mechanism for APCI.

Figure 1 highlights the mechanism for molecules to ionize through a gas-phase ion–molecule reaction as in APCI. When nitrogen gas is used, nitrogen plasma ionization is initiated by electrons, produced from a corona discharge needle. Ionized nitrogen plasma results in N_2_^+•^ or N_4_^+•^ and via charge transfer, N_4_^+•^ will ionize analytes with a low ionization potential. And since this process occurs at atmospheric pressure, water at the ion source is highly likely. H_2_O^+^ formed at the source reacts with another water molecule and produces H_3_O^+•^and this ion further reacts to produce water clusters (H_3_O^+^(H_2_O)_n_). These water clusters are also known to ionize the analyte molecule via proton transfer, thus promoting the protonated precursors [M + H]^+^. When comparing APCI and EI ion sources in GC-MS, they both perform similarly in reproducibility, dynamic range, limit of detection, and quantification. The main difference between the two is APCI’s ability to readily produce molecular ions, which are not commonly observed during EI. While obtaining a mass spectrum with molecular ion data is valuable, scientists have continuously opted out of using it due to the lack of availability of commercial mass spectral libraries. Thus, the main advantage of using GC-EI-MS is the availability of such reference data which can be used to identify components in question. While the implementation of EI for the identification of known drugs has been very successful, the rise of designer drugs that have not been cataloged requires a new approach, one that provides not only fingerprint data but also the mass of the protonated molecular ion. Thus, we believe that APCI libraries containing precursor and fingerprint data will be required for the rapid and efficient analysis of new and emerging designer drugs as they continue to be a problem.^53,54^ Therefore, this paper aims to introduce an APCI-GC-MS method capable of analyzing a wide range of fentanyl analogs of varying masses and chemical composition with the intention to be used alongside screening tools, such as DART-MS or Raman spectroscopy. Here we provide chromatographic and mass spectral data obtained using a triple quadrupole mass analyzer by varying collision energies in the second quadrupole for 74 fentanyl analogs. Also analysis of fragmentation tendencies of these moieties was conducted with the goal of information to aid in characterizing unknown fentanyl analogs.

## Experimental Section

### Materials

Currently, ∼212 fentanyl analogs have been detected, characterized, and cataloged by companies such as Cayman Chemical. The Fentanyl Analog Screening Kit and Emergent Panels 1, 2, 3, and 4 were obtained from Cayman Chemical. A list of the fentanyl analog standards and their respective mass and chemical formula analyzed in this paper as well as the mass spectral data are in Supporting Information Table S1. HPLC-grade methanol was purchased from Fischer Scientific. The reference standards and high-quality caffeine were purchased from Sigma-Aldrich. The majority of the fentanyl analogs studied here contained substitution at the *N*-alkyl chain, amide group, or aniline ring (see Figures S1–S6 in the Supporting Information for the structures).

### Sample Preparation

The Cayman chemical fentanyl samples were reconstituted with HPLC-grade methanol to obtain 1 mg/mL solutions. Fentanyl analogue mixtures containing 3–6 fentanyl analogs (of differing masses) with caffeine as an internal standard were prepared by adding the necessary volume of stock (1 mg/mL fentanyl sample solution) to HPLC-grade methanol to create a final solution of 10 ppm. Caffeine was added to the same vial as the internal standard to have a final concentration of 2 ppm.

### Instrumentation

APCI-GC-MS analysis was performed using an Agilent 7890B GC instrument for chromatographic separation and a Waters Xevo TQD Triple Quadrupole MS attached to a Waters Atmospheric Pressure Gas Chromatography (APGC) source that enables atmospheric pressure chemical ionization. The capillary column used was a DB5-MS (30 m length, 250 μm inner diameter, 0.25 μm film thickness). The carrier gas used was ultrahigh purity helium (99.999%) at a flow rate of 2 mL/min. The inlet temperature was 260 °C. The transfer line temperature was 280 °C. The oven temperature initially was 100 °C, and with injection, a ramp rate of 3 °C/min was immediately started until a temperature of 300 °C was reached and held for 3 min. An MS method was created and set to scan the specified precursor ion mass and to conduct subsequent CID by implementing collision energies of 10, 20, 30, 40, and 50 V. APCI and positive mode was used with a corona discharge needle current of 3 μA and voltage of 4 kV. The source temperature was 150 °C. The makeup gas was N_2_. The third quadrupole was set to scan from *m*/*z* 40 to M+20 of the desired precursor ion. This method ran for 64.5 min with a scan time of 0.1 s.

### Analysis and Data Processing

Qualitative Masslynx software 4.1 was used for APCI-GC-MS control, mass spectral peak integration, and mass spectra evaluation, as well as to extract GC retention times for all 74 analyzed compounds. The relative retention time (rrt) for each analyte was calculated by dividing each individual analyte’s retention time (rt) by an internal standard (caffeine) retention time (rt). Retention and relative retention time values are listed in Table S2 in the Supporting Information. MSMS data obtained from implementing different collision energies (10, 20, 30, 40, 50 V) was plotted using Origin 2023b with *m*/*z* values on the *x*-axis and relative intensity on the *y*-axis. Peak detection with a 30% threshold was implemented, and all data that met such threshold was tabulated.

## Results and Discussion

**Figure 2:**
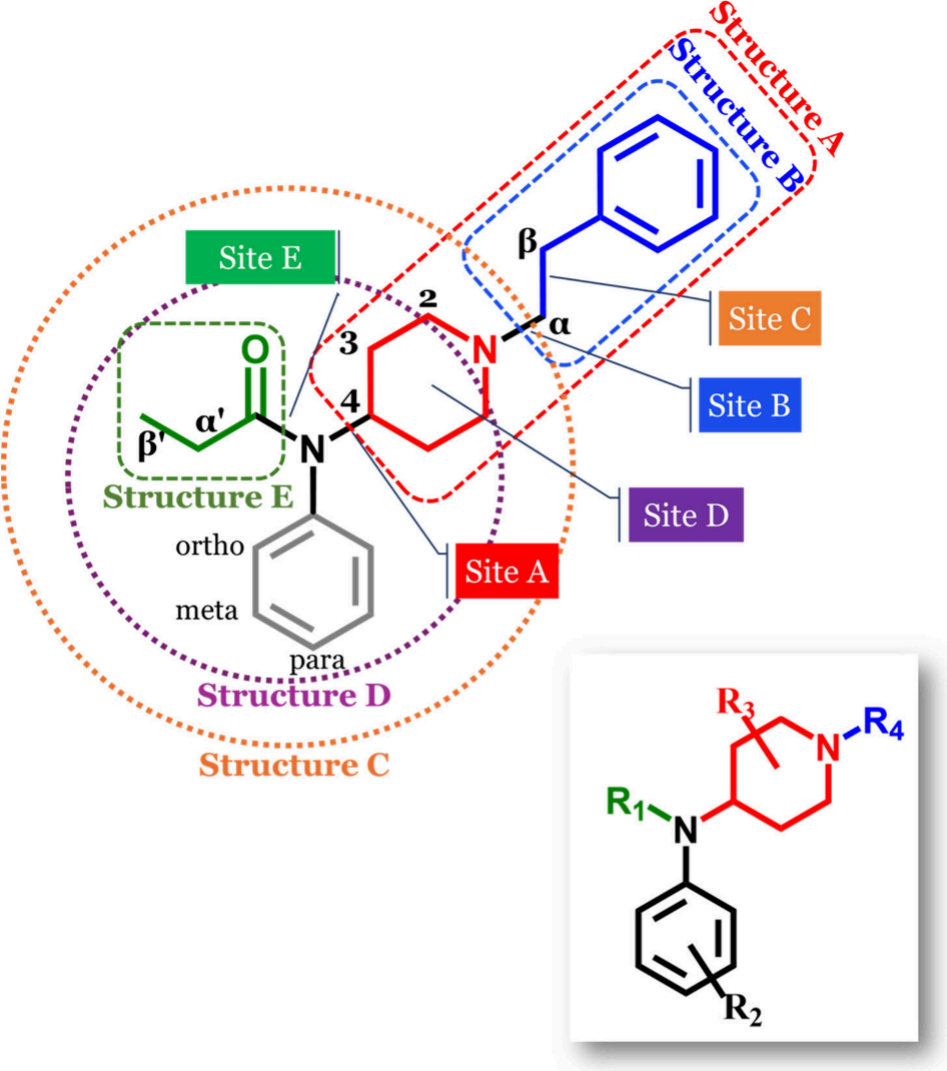
Fentanyl scaffold based off Cayman’s standardized naming convention. Cleavage at site A (the N–C4 bond) yields structure A. Cleavage at site B (the N-αC bond) results in structure B. Cleavage at site C (the αC-βC bond) produces structure C. Cleavage at the piperidine (site D) with subsequent loss of nitrogen produces structure D. Finally, cleavage at site E (the CO-N bond) generates structure E1.

Our mass spectral data show that the fentanyl compounds analyzed in this study typically fragment at five major sites: the amide N–C_4_ bond, the amide N-αC bond, the αC-βC bond, the piperidine ring, and/or the CO-N amide bond. We refer to these bonds as sites A-E, respectively, as illustrated in Figure 2. Fragmentation of the N–C_4_ bond (site A) generates product ion A, which consists of the piperidine ring base, along with the R_4_ group, which may be the commonly observed phenethyl moiety or any other N-terminus. In contrast, fragmentation at the N-αC bond (site B) produces product ion B, which consists solely of the N-terminal moiety (R_4_). Cleavage of the αC-βC bond (site C) results in product ion C, which consists of the tertiary amide group, including any modification at R1, R2, and R3. Fragmentation at site D, involving bond cleavage at any two points within the piperidine ring, results in structure D, which comprises the acyl group (R_1_), the aniline ring (R_2_), and a segment of the piperidine ring. Finally, fragmentation of the amide CO-N bond leads to the formation of structure E1 which consists solely of the acyl group (R1), or E2 which consists of the aniline and the piperidine ring moiety.

Product ions A-E, generated from initial bond cleavage at sites A-E, are primary fragments. All other fragments are subsequent fragments of these primary product ions and are thus termed secondary and tertiary fragments. Analogs can follow more than one fragmentation pathway, but there is only one pathway that it strongly follows, referred to as its primary fragmentation pathway. Fentanyl analogs that were seen to initially and primarily fragment at the amide site A, resulting in the abundant production of structure A at low CID energies (10–30 V), are classified as pathway A compounds. It should be noted that increasing CID voltages does not alter the primary fragmentation pathway designation. At higher energies instead of a new pathway, there are just fragments produced from the primary structure A. For example, if the primary pathway is producing compound A at voltages from 10 to 30 V, then at higher voltages of 40 and 50 V, compound B is being generated from fragmentation of compound A not via pathway B.

Analogs that initially and primarily fragment at site B, yielding structure B, or at site C, resulting in structure C, are classified as following pathway B/C. This combination of classifications is due to the simultaneous production of product ions B and C by these compounds. Compounds that primarily fragment at site D, producing structure D in abundance, are classified as pathway D compounds, while those that predominantly fragment at site E are designated as pathway E compounds. In this paper, we often refer to these compounds collectively as pathway A, B/C, D, or E analogs.

**Figure 3:**
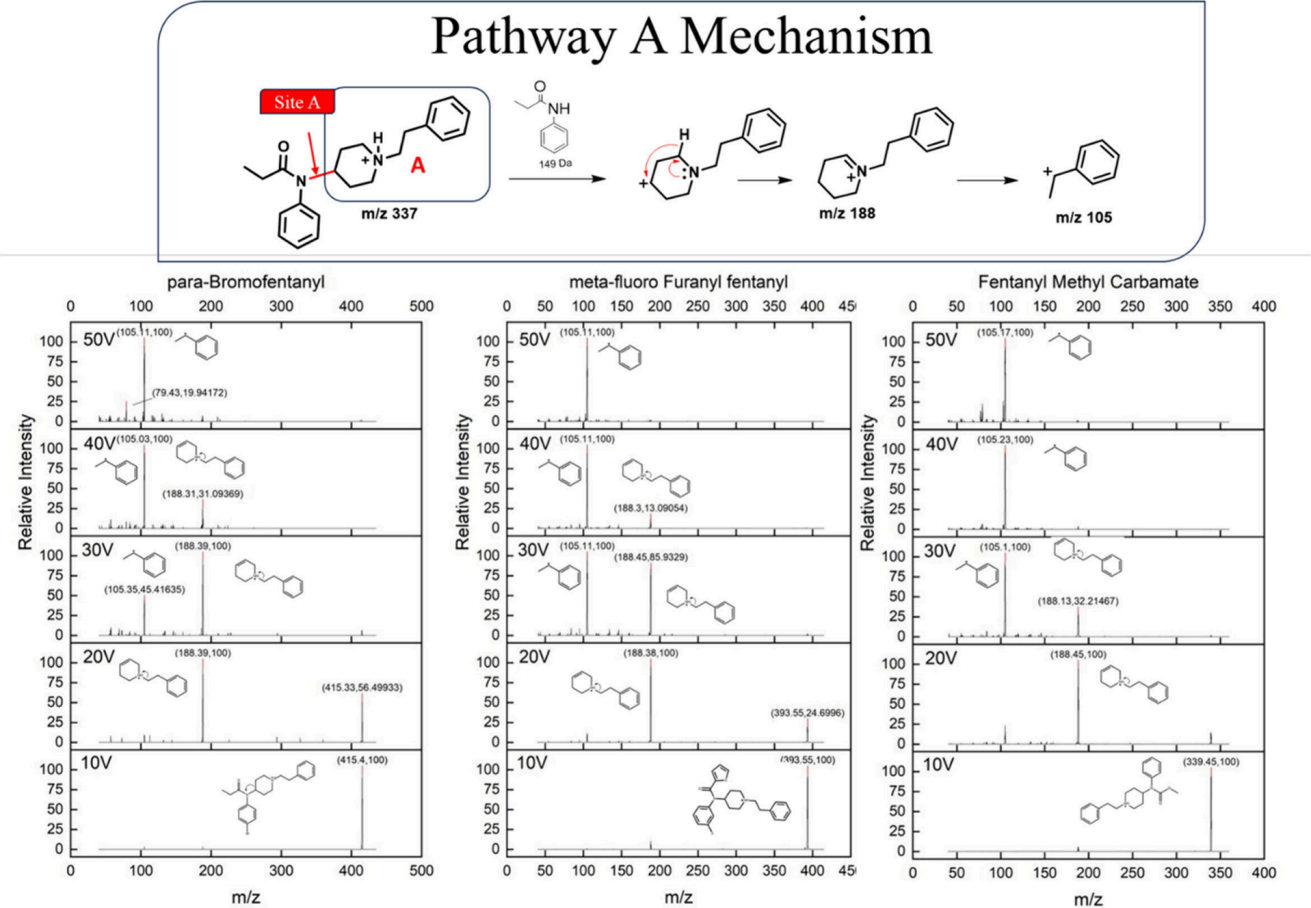
Illustration depicting fentanyl (*m*/*z* 337) undergoing dissociation via pathway A. Fragmentation occurs at the amide N–C4 bond, yielding product ion A (*m*/*z* 188), which further fragments to produce product ion B (*m*/*z* 105). Additionally, the figure presents the mass spectra for para-bromofentanyl, meta-fluoro furanyl, fentanyl, and fentanyl methyl carbamate, all of which also undergo pathway A dissociation.

Of the 74 analogs analyzed in this study, 31 (45%) contain modifications at either R1, R2, or R3 or in combination. R1 groups are either acryl, acetyl, linear, or branched alkyl chains, methoxy groups, cycloalkanes, aromatic rings, or heterocyclic rings. If an R2 functional group is present at the aniline ring, it is either a single fluorine, bromine, or chlorine heteroatom. If an R3 functional group is present, then it is a single methyl. Figure 2 depicts several of the fentanyl analogs that contain modifications at R1, R2, or R3. Additionally, 39 compounds, which make up 57% of the sample pool, contain modifications solely at R4 or in combination with other modifications at the other sites mentioned, as discerned from Figure 3. R4 modifications typically involve the addition of functional groups to the phenethyl moiety or the complete substitution of the N-terminus with groups like dihydrobenzofuranyl, and only 4 analogs lacked R1 and thus contained no acyl group.

Overall, as depicted in Figure S7, of the 74 fentanyl analogs analyzed, 40 (54%) primarily fragment at site A, 30 (41%) primarily fragment at site B and/or C, and 4 (5%) primarily fragment at site D. Tables S3, S4, and S5 in the Supporting Information list the fentanyl analogs that pertain to pathways A, B/C, and D, respectively. Furthermore, APCI-GC-MS readily produces [M + H]^+^ species for all 74 fentanyl analogs, and for those analogs that remain unmodified at the piperidine ring and feature a phenethyl moiety as their R4 group, induced dissociation typically yields mass-to-charge ratios of 188 and 105, corresponding to structures A and B, respectively. These *m*/*z* values are widely recognized as reliable indicators of the presence of a fentanyl derivative in a sample. Product ion *m*/*z* 188 (C_13_H_18_N) is obtained via pathway A. This primary product ion subsequently fragments at the piperidine N-αC bond, resulting in the loss of the piperidine ring as a neutral species and producing a secondary product ion with an *m*/*z* of 105 (C_8_H_9_). Each pathway is individually discussed in more detail in subsequent sections.

### Pathway A

Of the 74 analogs analyzed, 40 (54%) adhere to pathway A, characterized by primary bond cleavage at the N–C4 bond and a high abundance of product ion A, as depicted in Figure 3. Among these 40 compounds, 26 (65%) feature substitutions at either the amide group (R1), the aniline ring (R2), or positions C3–C5 of the piperidine ring (R3). Additionally, 9 compounds (22%) include at least one functional group on the phenyl ring of the *N*-alkyl chain, while 3 compounds (7%) possess multiple functional groups on the phenethyl moiety. Notably, 3 (7%) compounds lack an R1 group, classifying them as despropionyl-based fentanyl analogs. It is important to emphasize that although these analogs predominantly fragment through pathway A, this is not their sole route. A small percentage (approximately 8%) of pathway A analogs also proceed via pathway E, resulting in the formation of structures E1 or E2. We will explore these analogs in greater detail later in the paper.

For these 40 analytes, the molecular ion [M + H]^+^ typically emerges as the base peak at 10 V, as annotated in Table 1 showing a representative select number of analytes (for a complete list, please refer to Table S3). However, there were a limited number of compounds that generated a product ion in addition to the parent ion ([M + H]^+^) at such a voltage. Despropionyl meta-methylfentanyl, despropionyl 2′-fluoro ortho-fluorofentanyl, and 4-methyl fentanyl all produced ion A at low concentrations at 10 V, roughly making up 30% of the base peak. Notably, these analogs lack an R1 group in the acyl region, thus increasing the amide nitrogen affinity for a proton and thus affinity for protonation. 4-methyl fentanyl was the sole analyte to produce product ion A at an intensity of 90% of the base peak at 10 V. The presence of a methyl group at C4 of the piperidine ring increases steric hindrance in that region, thereby promoting fragmentation at site A and destabilizing the molecular ion at weaker voltages. We did not analyze any other analogs with a functional group at C4, so we cannot definitively conclude that such analogs will behave similarly. However, we encourage readers to consider the presence of product ion A at 10 V as a potential indicator of structural instability in the amide region, or a lack of an acyl carbon.

**Table 1:**
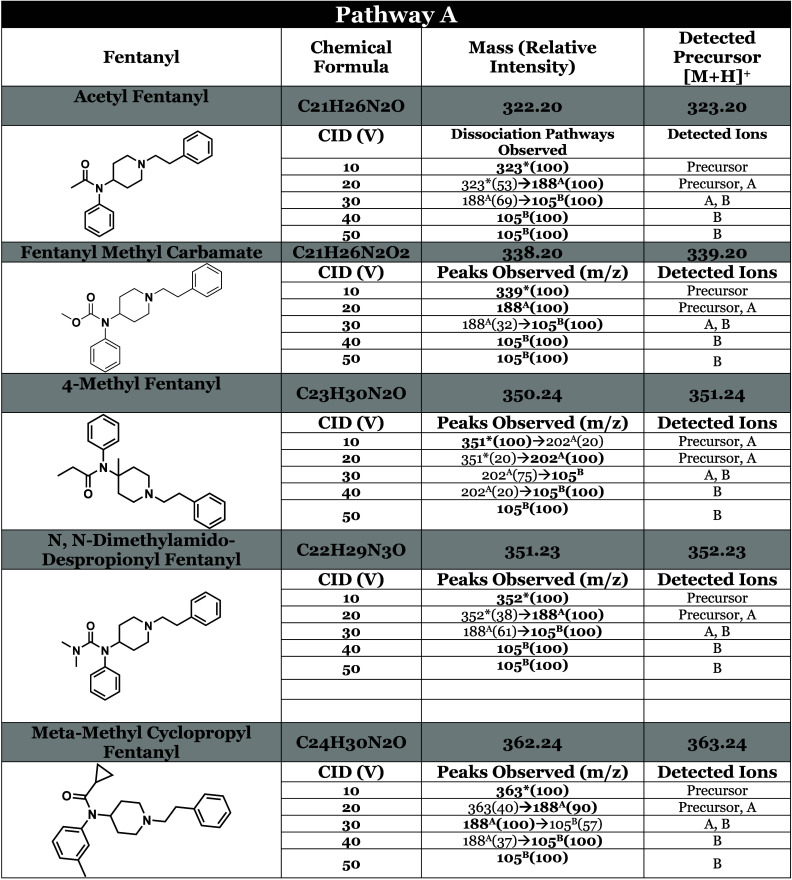
Tabulated MS/MS Data for 5 Examples of Fentanyl Analogs That Primarily Fragment at the N–C4 Bond (Site A), Resulting in a High Abundance of Product Ion A at Low Collision Energies (10–20 V)^a^

a All compounds that follow pathway A are listed in Table S1 of the Supporting Information. Precursor ions are marked with an asterisk (*), base peaks are bolded, and superscript annotations indicate the following cleavage sites: (#^A^) cleavage at the N–C4 bond, (#^B^) cleavage at N-αC, (#^c^) cleavage at αC-βC, (#^D^) cleavage at the piperidine ring, and (#^E^) cleavage at the CO-N amide bond, relative intensities are denoted in parentheses.

Many of these compounds predominantly display product ion A as the base peak at 20 V, and occasionally at 30 V. At higher voltages (40–50 V) we observe subsequent fragmentation of product ion A. If the fentanyl analog has an unmodified piperidine ring along with an ethyl alkyl chain, then subsequent fragmentation of A produces structure B with a *m*/*z* of 105. Since several of these analogs remain unmodified at the piperidine ring and phenylethyl moiety, product ion A is consistently assigned to the *m*/*z* 188 peak (C_13_H_18_N). Additionally, these compounds often produce ion B, the subsequent fragment of A, as the base peak at both 30 and 40 V, which is generally associated with the *m*/*z* 105 peak (C_8_H_9_). When there is a highly stabilized R1 group (generally these are highly conjugated acyl groups), fragmentation shifts to occur more readily at site E. Thus, we see a higher production of product ion E in our mass spectra in comparison to analogs that do not have highly stable R1 groups. Furthermore, while most pathway A compounds that generate structure A as the base peak at 20 or 30 V contain only a single functional group at the phenyl moiety, a few compounds deviate from this trend. These compounds N-(2C-TFM) fentanyl, N-(2C–I) fentanyl, and 2′,5′-dimethoxy fentanyl are highly substituted at the phenyl terminus, each containing at least two methoxy functional groups. Thus, they consequently resemble other analogs that are extensively substituted at the *N*-alkyl chain but dissociate via pathway B or C. Therefore, we would expect their behavior to align with this pattern, yielding A at intensities at or below 50% of the base peak ([M + H]^+^), yet this is not the case. As annotated in Table S1, these analogs generate A as the base peak at 20 or 30 V, thereby following pathway A. However, due to the structural similarities among these three analytes, they will be discussed further in the subsequent section focusing on pathway B/C analogs.

### Pathway B and C

**Figure 4:**
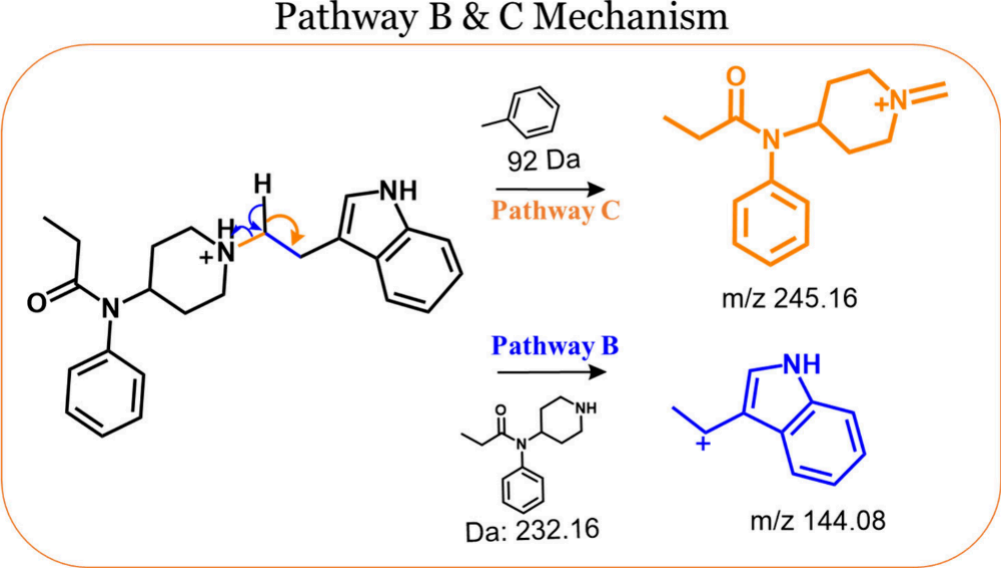
Depiction of N-(3-ethylindole) norfentanyl undergoing dissociation via pathways B and C. For pathway B, the amide N-αC bond is fragmented, resulting in a charged product ion B (*m*/*z* 144), while fragmentation at site C produces product ion C (*m*/*z* 245) as the charged species.

Thirty (40%) of the 74 fentanyl analogs analyzed in this study preferentially fragment at sites B or C of the *N*-alkyl chain during low-collision energy scans (10–20 V), resulting in the production of product ions B and C (see Table S4 in the Supporting Information to form a complete list of compounds). As depicted in Figure 4 for N-(3-ethylindole) norfentanyl, fragmentation at the N-αC bond produces a structure B that includes the *N*-alkyl chain, while fragmentation at the αC-βC bond produces a structure that incorporates the tertiary amide group. As illustrated in Figures S8 and S9 (in the Supporting Information), the R1, R2, and R3 groups in these structures are unmodified, resulting in identical tertiary amide groups. However, all of these analogs contain multiple functional groups along the *N*-alkyl chain, suggesting that highly substituted fentanyl analogs are likely to fragment via pathway B or in combination with pathway C.

Unlike pathway A analogs, which primarily produce product ion A as the base peak at both 20 V and occasionally at 30 V, these 30 compounds in question never produce A as the base peak. Instead, A is typically not present, but when it is detected, its intensity is either similar to that of ion B and/or C or at considerably lower intensities than either ion. As can be discerned from Table S1, for 11 of the 30 compounds, structure B is the only product ion detected at 20 V, with relative intensities that vary significantly, ranging from about 30% to 90% of the base peak ([M + H]^+^). As seen in Figures S4 and S5 (in the Supporting Information section), a notable trend among these analogs with the exception of 2′,4′-dimethoxy fentanyl that exclusively produce ion B is the presence of three functional groups at the phenyl terminus, two of which are methoxy groups. The third functional group at the para position is either a linear or branched alkyl chain, a linear alkylthio group, or a halogen (such as Br or I). Another significant trend is that over half of these compounds feature a methyl group at the α carbon of the alkyl chain. Methoxy groups are recognized for their strong electron-donating properties through resonance, while also exhibiting a weak electron-withdrawing inductive effect. Therefore, we consider that the electronic characteristics of these methoxy groups create sufficient electron pull on the phenyl electron cloud, leading to a slight positive charge on the phenyl ring. This, in turn, enhances the ring’s tendency to inductively attract electrons from the alkyl chain, thereby weakening the N-αC bond of the piperidine; and the introduction of highly electronegative halogens to the phenyl moiety further amplifies this electron-pulling effect on the alkyl chain. We also hypothesize that the methyl group at the α carbon increases steric hindrance in that region, promoting preferential dissociation at this bond and resulting in the formation of more stable product ion B.

Furthermore, five of the 30 pathway B analogs produce comparable amounts of product ions A and B at 20 V. These analogs include N-(2C–D) fentanyl, N-(2C-T-2) fentanyl, 2′,3′-dimethoxy fentanyl, 2′,6′-dimethoxy fentanyl, and N-(2C-E) fentanyl. All these five analogs lack a methyl group at the α carbon of the *N*-alkyl chain, and when present, the third functional group on the phenyl moiety—aside from the two methoxy groups—consists of shorter versions of the alkyl and alkylthiol linear chains observed in the compounds that exclusively produced product ion B. This supports the idea that the presence of the methyl at the α carbon is a major driving force in the instability of the N-αC bond and thus in the production of ion B. It is important to highlight that 2′,3′-dimethoxy fentanyl, 3′,4′-dimethoxy fentanyl, 2′,6′-dimethoxy fentanyl, and 2′,4′-dimethoxy fentanyl, each of which belongs to pathway B, produce varying intensities of ions A and B. While 2′,6′-dimethoxy fentanyl and 2′,3′-dimethoxy fentanyl produces A and B at comparable intensities at 20 V, 3′,4′-dimethoxy fentanyl generates only ion A at this voltage, and 2′,4′-dimethoxy fentanyl does not produce structure A at any of the voltages. Apart from these 4 analogs, another dimethoxy isomer exists (2′,5′-dimethoxy fentanyl) but is categorized as a pathway A analogue due to it producing structure A as a base peak at 20 V. In attempting to understand the difference in production of A vs B for these dimethoxy based analogs, we conclude that computation modeling is required for further understanding of such behavior, a topic outside of the scope of this paper.

Furthermore, when comparing the relative intensities of structure A at 20 V for fentanyl analogs featuring a halogen at the para position of the phenyl ring, a clear trend emerges relating the electronegativity of the halogens to the intensity of the produced ion A. As the electronegativity of the heteroatom increases, the amount of produced A decreases while that of ion B increases. For instance, N-(2C–C) fentanyl, which contains a chlorine atom at the para position of the phenyl ring, produces only ion A at 20 V. Its subsequent fragment (*m*/*z* = 199) emerges as the base peak at 30 V, indicating that A is highly unstable and rapidly undergoes dissociation at the N-αC bond. In contrast, N-(2C–B) fentanyl, which features a bromine atom at the para position of the phenyl ring, begins producing ion A at 20 V, with intensity increasing as the voltage rises to 30 V, thus indicating that its A structure is more stable than the latter. As for N-(2C–I) fentanyl, it produces A as the base peak starting at 30 V, thus indicating that the N-αC bond is stronger for this compound compared to those of the other two. This behavior can be explained by examining the inductive withdrawal effect exerted on the *N*-alkyl chain by the attached functional groups on the phenyl moiety. Electronegative atoms (Cl, Br, and I) tend to attract electrons toward themselves through induction, resulting in a partial negative charge on the halogen and a corresponding slight positive charge on the phenyl ring. Less electronegative atoms exert a weaker pull compared to their more electronegative counterparts. Consequently, chlorine pulls electrons more strongly than bromine, which, in turn, pulls more strongly than iodine. This electron withdrawal makes the phenyl ring more susceptible to attracting electrons from the attached alkyl chain, thus weakening the N-αC. Therefore, if ion A is suspected to undergo rapid dissociation into its subsequent fragment, especially when compared with another molecular ion, this may indicate the presence of a more electron-withdrawing functional group.

Of the 30 pathway B/C compounds, only ten produced product ion C, and they did so only in combination with ion A and/or B. As shown in Figures S4, S5, and S9, the N-terminus of these compounds either is highly substituted with strong electronegative functional groups or features a highly stabilized moiety in place of the base phenyl group. Analogs featuring a highly stabilized R4 group, such as an indole or benzofuranyl group—showed in N-(3-ethylindole) norfentanyl and N-(2-APB) fentanyl—or a highly electronegative functional group like a nitro group, as seen in N-(2C–N) fentanyl, were observed to produce structure C as the base peak at 20 V. In the case of N-(6-APDB) fentanyl and N-(MDA) fentanyl, which also feature highly stable aromatic ring systems at the N-terminus, ion C is not produced abundantly. Instead, it appears at approximately 30% of the intensity of the base peak, which is either the molecular ion or product ion B.

We believe that the significant difference in the production of ion C can be attributed to the distance between the alkyl chain and the highly electronegative atoms in the R4 group. In N-(6-APDB) fentanyl and N-(MDA) fentanyl, the alkyl chain is attached to carbons 6 and 5 of the dihydrobenzofuranyl and benzodioxolyl structures, respectively. As a result, the oxygen atoms are not in close proximity to the alkyl chain, unlike in N-(3-ethylindole) norfentanyl and N-(2-APB) fentanyl. This trend regarding the electronegativity of the attached functional groups also applies to the other pathway B/C compounds. N-(3,4,5-TMA) fentanyl features a third methoxy group at its N-terminus, enhancing the electron-withdrawing effect and resulting in the production of ion C at higher intensities (∼80%). In contrast, analogs with a less electronegative third functional group, such as thio-, methyl, or chlorine—seen in N-(DOC) fentanyl, N-(2C-T-4) fentanyl, and N-(2C-G) fentanyl—produce ion C at only 30% of the base peak, which is either the molecular ion or product ion B. We conclude that R4 groups that are either highly stabilized or contain highly electronegative functional groups that exert electron-withdrawing inductive effects will promote fragmentation at both site C and site B. However, the intensity of ion C observed is influenced by the electronic effects of the substituents. It is also important to note that while a highly stable aromatic ring, such as benzofuranyl, may be present at R4, its attachment to the alkyl chain plays a crucial role. For significant inductive effects that lead to the production of ion C, the electronegative atoms in the ring must be directly linked to the alkyl chain.

Furthermore, as previously mentioned, there are two outliers that diverge from the trends observed in both pathways A and B/C. Both N-(2C-TFM) fentanyl and N-(2C–I) fentanyl feature two methoxy functional groups on the phenyl moiety, along with highly electronegative halogens (I and CF_3_) at the para position. Given the presence of these electronegative atoms, we would expect these compounds to produce product ion B at intensities much higher than that of A. However, as shown in Table S1, both analytes produce A as the base peak at 30 V, indicating that A is highly stable at this voltage. This finding contradicts the trends observed in the other 72 fentanyl analogs, and thus caution should always be taken when assessing these *m*/*z* values and intensities.

### Pathway D

**Figure 5:**
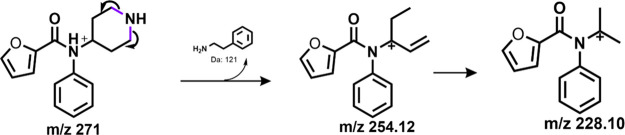
Furanyl norfentanyl undergoes fragmentation via pathway D, which consists of the loss of phenyethylamine as a neutral while the charge is retained on the amide moiety.

Among the 74 analogs analyzed in this study, only 4 (Table 2) exhibited fragmentation at bonds N–C2 and N–C6 of the piperidine ring, leading to the neutral loss of the amine nitrogen, resulting in an open ring system as shown in Figure 5 for furanyl norfentanyl. Notably, although 2,3-seco-fentanyl, listed in Table 2, lacks a piperidine ring, fragmentation at the tertiary amine still results in the loss of the phenylethylamine moiety.

**Table 2:**
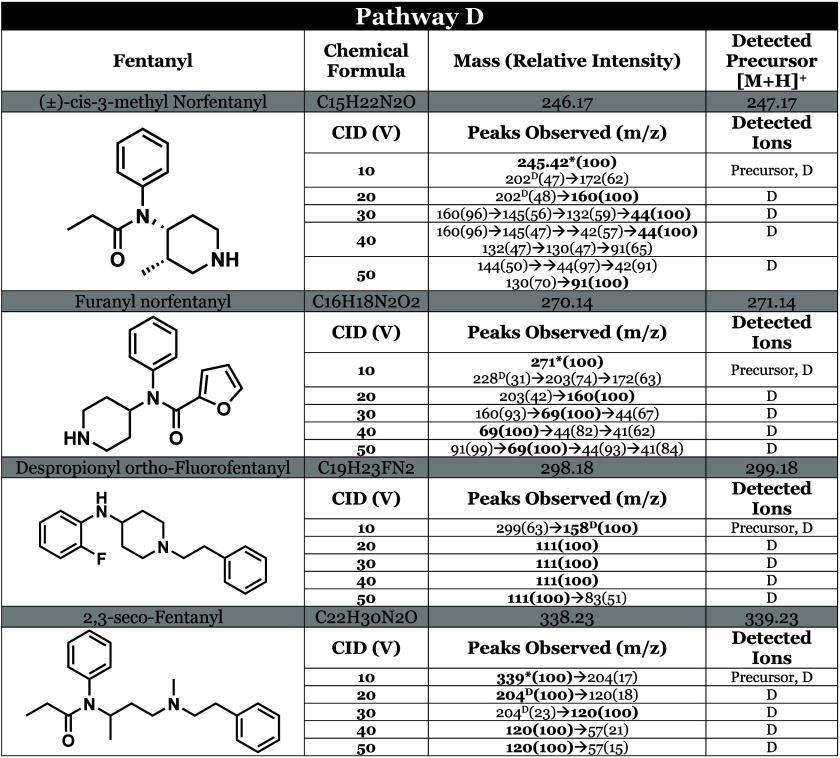
Tabulated MSMS Data of the Fentanyl Analogs That Follow Pathway D^a^

a All compounds that follow pathway D are listed in Table S1 of the Supporting Information. Precursor ions contain an asterisk (*), base peaks are bolded, superscript (#^A^) indicates the *m*/*z* value was obtained by cleavage at the N–C4 bond, (#^B^) cleavage at N-αC, (#^c^) cleavage at αC-βC, (#^D^) cleavage at piperidine ring, and (#^E^) cleavage at CO-N amide bond, relative intensities are denoted in parentheses.

As seen in Table 2, despropionyl ortho-Fluorofentanyl is one of the few analogs that preferred to fragment at the piperidine ring, thus following pathway D. One would assume that isomers of the analogue would follow the same fragmentation pathway, but our data concludes otherwise. Despropionyl para-Fluorofentanyl is an isomer of the latter which follows pathway A with no evidence of pathway D occurring. The only possible explanation of the difference in pathways taken is the position of the fluorine, but computational analysis is required to prove that fluorine’s position on the phenyl ring dictates whether an analog will undergo pathway D fragmentation. As for (±)-cis-3-methyl norfentanyl and furanyl norfentanyl, the unstable nature of the secondary amine and the structures’ ability to rearrange into a stable multicyclic structure with the loss of NH_3_ explains why pathway D is favorable.

### Pathway E

**Figure 6:**
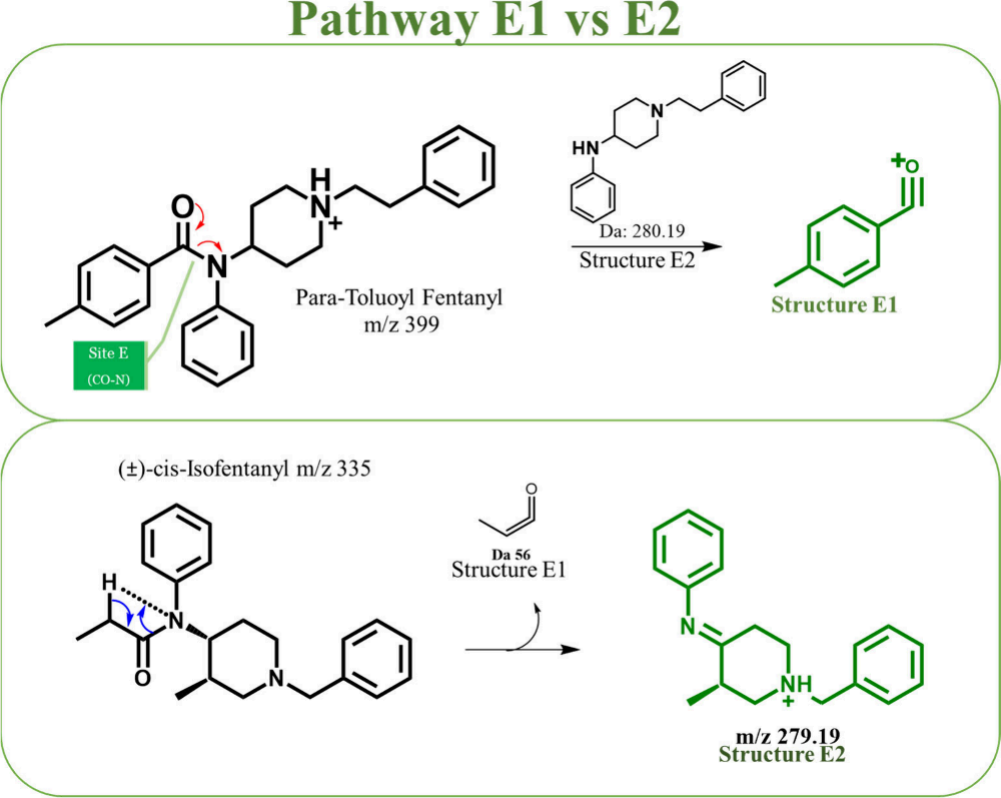
Mechanisms of pathway E1 (top) depicting how para-toluoyl fentany fragments at site E result in the formation of a charged acyl product ion, E1. In contrast, pathway E2 (bottom) involves the loss of the acyl group (E1) as a neutral species, leading to the generation of product ion E2 as the charged species.

Of the 74 fentanyl analogs studied, only eight compounds were observed to follow pathway E. Specifically, seven compounds yielded structure E1 while only one produced structure E2. These product ions (E1/E2) result from fragmentation at the amide CO-N bond. As depicted in Figure 6, product ion E1 is a positively charged acylium ion which provides information regarding the acyl group (R1), a region that is commonly modified in fentanyl derivatives, while product ion E2 is a protonated piperidine moiety.

The seven fentanyl analogs that generated product ion E1 include norsufentanil, senecioylfentanyl, tigloylfentanyl, para-toluoylfentanyl, para-chloro-furanylfentanyl 3-furancarboxamide, 2,3-benzodioxolefentanyl, and para-methyl cyclopentyl fentanyl. Their structures are illustrated in Figure S10 and the corresponding mass spectral data can be found in Table 3. The mass spectral data indicate that all of these seven compounds primarily produce product ion A at low CID voltages (10–20 V). At higher voltages, these compounds produce E1 as a base peak, and when not a base peak, the intensity of E1 is typically at or above 50% of the base peak intensity. This indicates that there is a shift in the primary fragmentation pattern for these analogs, specifically from pathway A to pathway E. Thus, we can say that pathway E is a competing pathway of A at higher CID voltages. As anticipated, the intensity of product ion E1 correlates with its structural stability; more stable R1 groups yield a greater and more persistent presence within the 20–50 V range. For instance, R1 groups containing highly reactive oxygen atoms (excluding the acyl carbon) dissipate more rapidly than their more stable counterparts. Notably, the intensity of the R1 group in 2,3-benzodioxolefentanyl is consistently lower than that of the R1 group in para-toluoylfentanyl. In the case of para-chloro-furanylfentanyl 3-furancarboxamide, the R1 group is detectable only at 30 V, likely due to the inherent instability of this functional group.

**Table 3:**
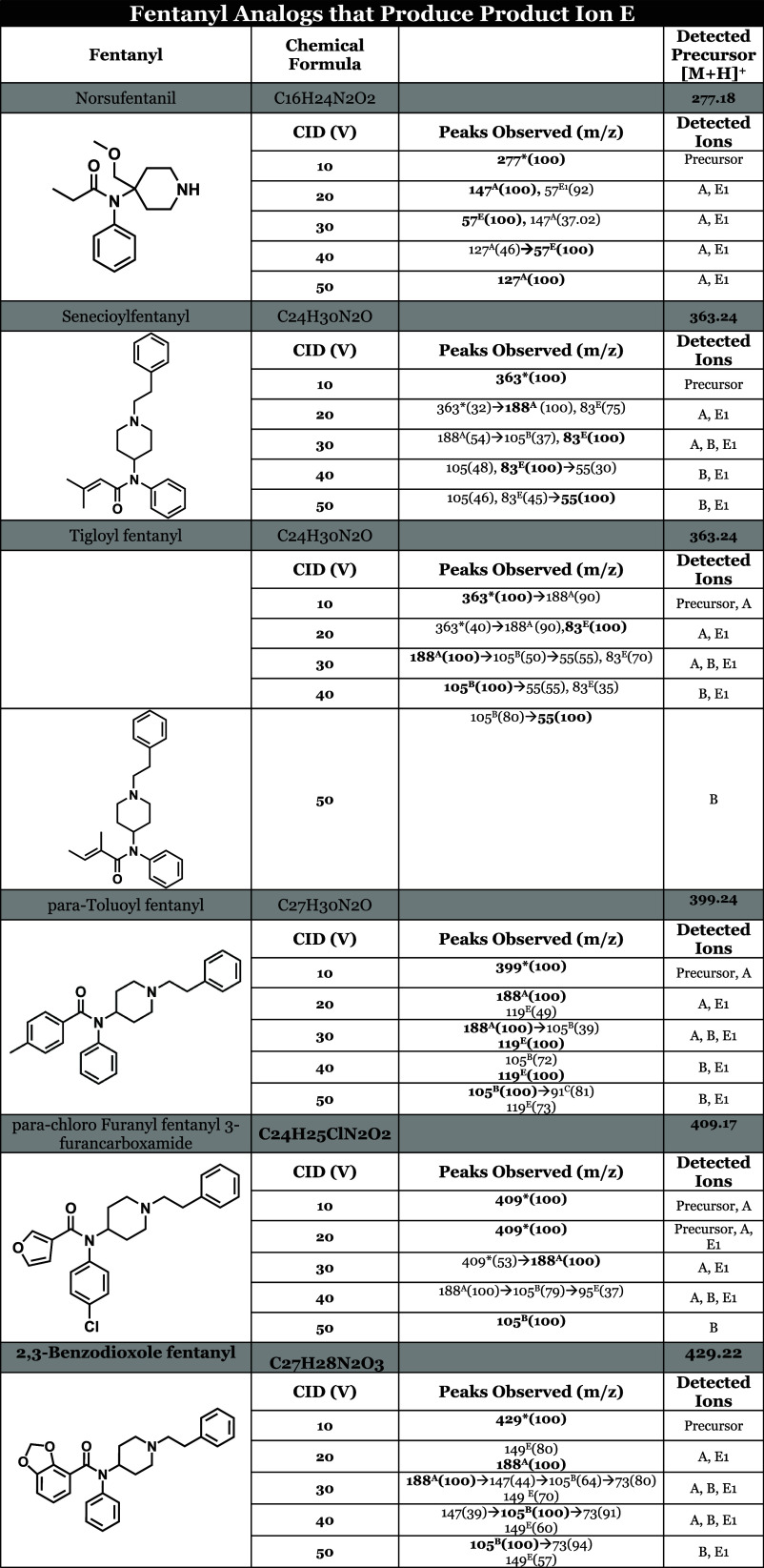

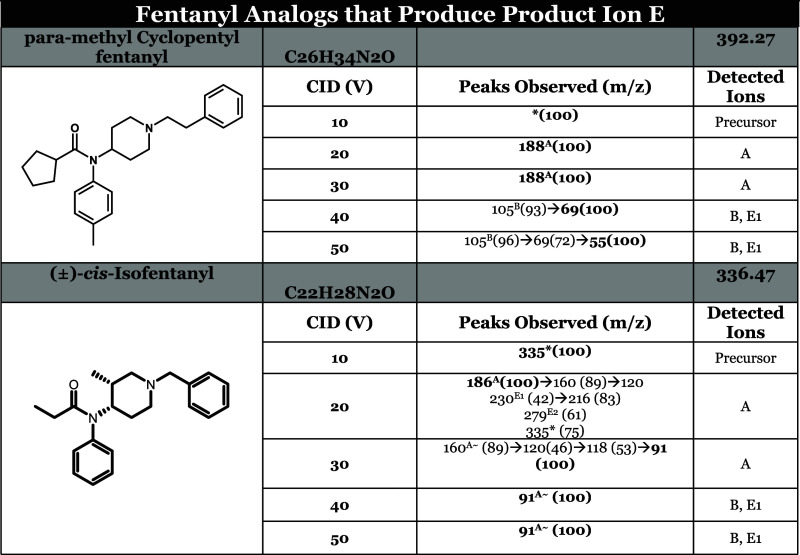
Tabulated MSMS Data for Examples of Fentanyl Analogs That Produce Structure E (Amide Group)^a^

a Precursor ions contain an asterisk (*), base peaks are bolded, superscript (#^A^) indicates the *m*/*z* value was obtained by cleavage at the N–C4 bond, (#^B^) cleavage at N-αC, (#^c^) cleavage at αC-βC, (#^D^) cleavage at piperidine ring, and (#^E^) cleavage at CO-N amide bond, relative intensities are denoted in parentheses, ∼ denotes subsequent fragment of primary fragment ion.

Of the 74 analogs analyzed in this study, (±)-cis-Isofentanyl was the only compound that produced structure E2. This compound primarily produced structure A and never produced structure E2 abundantly and was thus listed as a “pathway A analog”. As illustrated in Figure 6, structure E2 is generated by cleaving the amide CO-N bond (site E). In this process, the amide nitrogen abstracts hydrogen from the adjacent alkyl chain, resulting in the formation of product ion E2 rather than E1. For (±)-cis-isofentanyl, product ion E2 has an *m*/*z* of 279 with a relative intensity of 61%, and it consists of the aniline ring and the *N*-alkyl chain as the charged moiety. Notably, it is the only compound featuring a methyl alkyl linker and a methyl group at the C5 position of the piperidine ring. We suspect that due to the steric hindrance caused by this methyl group, the structure prefers to lose the amide group to free up space to become a more stable structure. Since we analyzed only one compound with these specific structural characteristics, we cannot draw general conclusions about whether this behavior is typical for similar compounds.

### Fragmentation vs Molecular Mass

We investigated the relationship between the mass-to-charge ratio (*m*/*z*) and the fragments observed and grouped them into categories by weight in increments of 50 *m*/*z*. As the precursors increased in weight from 250 to 300 to 301–350 *m*/*z* (see Supporting Information Figures S11 and S12), the precursor ion became more resilient to fragmentation and appeared in the mass spec at 20 V as opposed to only 10 V for the lower molecular weights. Product ion A was generally more stable with fragments at 20 and 30 V. Product ion B was visible in most compounds from 20 to 50 V of collision energy for all mass ranges (Figures S11–16) even when other fragments were not observed. Product ion C was not as stable as product ion B in the lower molecular weight groups but became more prominent as the molecular weight of the compounds increased. Deviation from pathway B occurred more often as the collision energy reached 50 V. While many compounds exhibited product ion B molecules at 50 V, there were also other peaks not associated with the typical pathways, giving rise to smaller fragments. APCI produced product ions A and B most readily, with the other pathways C, D, and E occurring less frequently.

### A Note about APCI-GC-MS Values vs EI-GC-MS Values

When comparing our fragments produced by APCI to the fragments from the Cayman chemical database produced by EI, the fragments from APCI tend to be more predictable with most of the fragmentation following pathway A and pathway B. The Cayman web site hosts a database containing 70 eV EI MS data of hundreds of emerging forensic drug standards. The data from the Cayman chemical database produced by electron impact shows more fragmentation than that produced by APCI; however, predicting the EI fragmentation pathways is not easy.^55−58^ For example, the electron impact of para-bromofentanyl like many other fentanyl analogs does not resemble the results obtained in our lab with APCI. Electron impact (EI), as a hard ionization source, ionizes the molecule of interest directly and can positively charge the molecule of interest, leading to fewer intact molecular ions. Atmospheric pressure chemical ionization (APCI) ionizes a reagent gas (N_2_) which gives the analyte a positive charge; this soft ionization method mainly generates precursor molecules. The two different ionization methods produce spectra that do not resemble each other with different base peaks and low to nonexistent relative intensities of other peaks. As seen in Figure S17, product ions E and C are more commonly observed in EI but product ions A and B are more common in APCI.

## Conclusion

**Figure 7:**
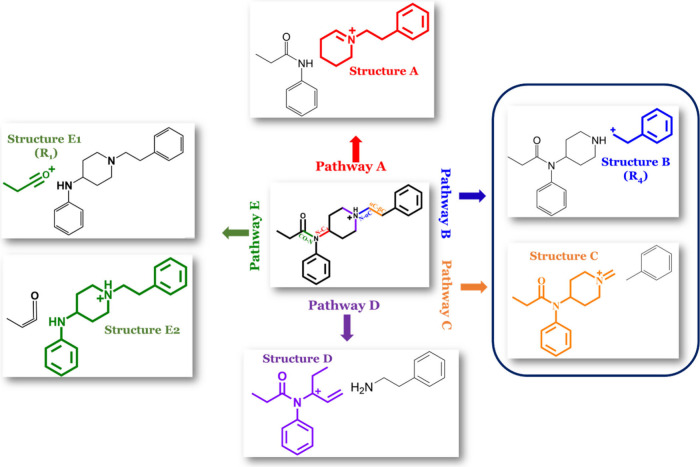
Illustration of product and neutral molecules produced by APCI-GC-MS. Pathways A and B/C, producing structures A and B, are the more prominent fragmentation pathways occurring in APCI. Pathways D and E are observed to occur in fewer of the molecules.

APCI-GC-MS instrumentation was used to analyze 74 fentanyl analogs at varying CID voltages of 10, 20, 30, 40, and 50 V. MSMS data for each analogue was successfully obtained and used for trend analysis. Our analysis indicates that the fentanyl analogs examined through APCI-GC-MS predominantly follow pathways A, B, and C, with pathways D and E (Figure 7) occurring less frequently. Analogs with one or more substituents at R1, R2, or R3 tend to fragment preferentially at site A. This fragmentation results in a high abundance of product A at voltages between 20 and 30 V, followed by the formation of product B, which becomes the base peak at 30–40 V. Fentanyl analogs featuring only a single substituent on the N-alkyl chain exhibit the same fragmentation pattern. In contrast, highly substituted fentanyl derivatives, particularly at the N-terminus, generally fragment along the N-alkyl chain. This leads to the predominant formation of structures B and C. Product ion A is typically observed as the base peak at low voltages and decreases in intensity at higher voltages for most fentanyls regardless of weight. In our analysis, we identified only seven analogs that produced product ion E1 and only one produced product ion E2. The acyl groups associated with the analogs that produced E1 were notably stable, including highly stabilized structures such as benzodioxole, phenyl rings, and furanyl rings. Therefore, if a stable enough acyl R1 group is present, fragmentation shifts from site A to site E; thus, it can be stated that pathway E is a competing pathway of A in these instances. Out of the 74 fentanyl analogs analyzed, only four yielded product ion D, and these were compounds that either lacked the R1 or R4 group.

**Figure 8:**
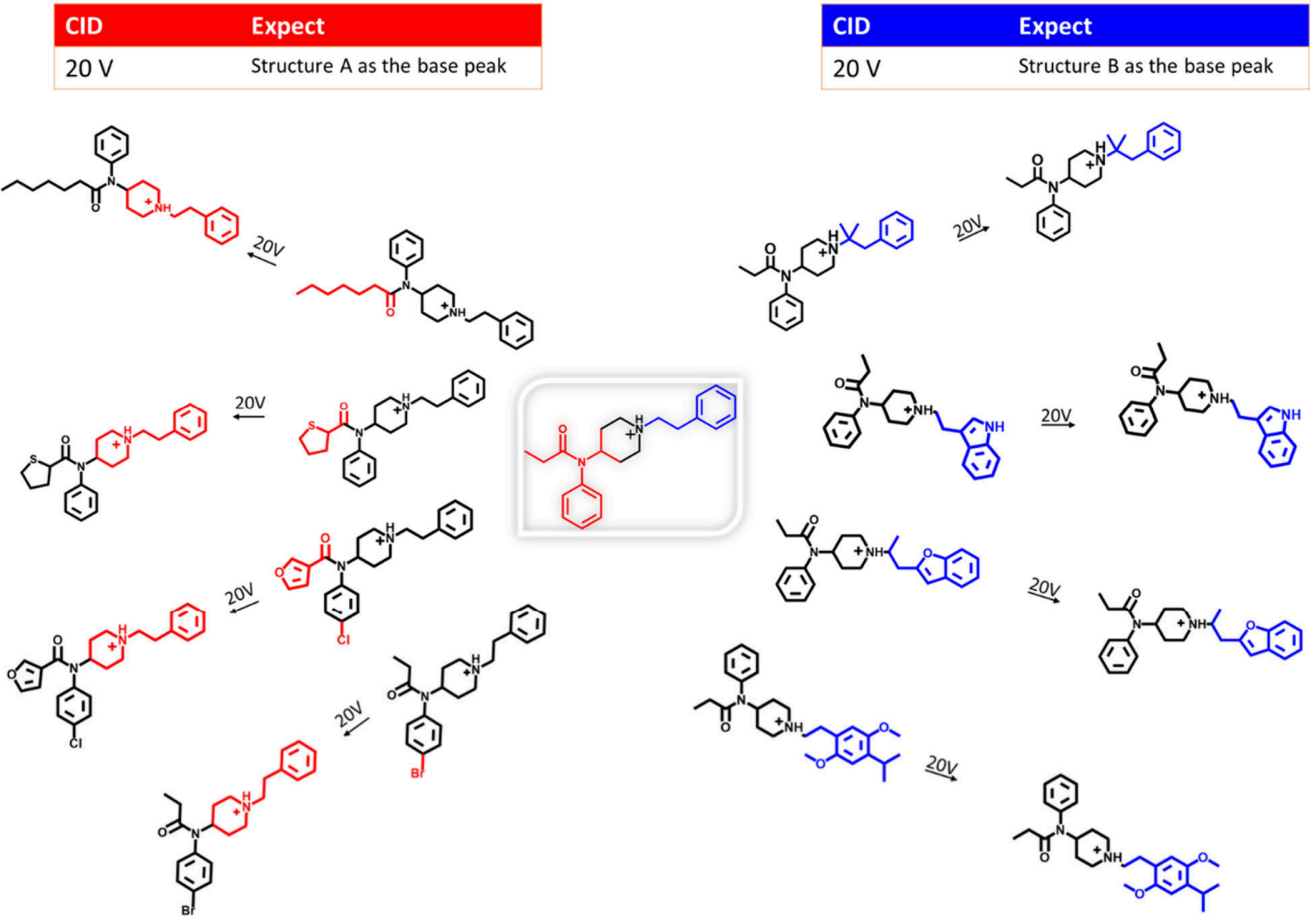
Illustration of expected product ions APCI at 20 V. If an analogue is highly substituted at the tertiary amine or C3–C5 of the piperidine ring, structure A is expected at the base peak at collision energy (20 V). If an analog is highly substituted at the N-alkyl chain, structure B is expected as the base peak as collision energy (20 V).

The wide range of collision energies (10–50 V) implemented in this experiment was sufficient to produce stable precursor ions as well as primary and secondary product ions. If a fentanyl structure is to be confirmed using APCI-GC-MS, and the proposed structure is highly substituted at the amide group or C3–C5 carbons of the piperidine ring, cautiously assume that the base peak at 20 V is a product ion (that includes the piperidine ring and N-alkyl chain moiety) produced from fragmentation at the N–C4 bond, as seen in Figure 8.

If the structure is instead highly substituted at the N-alkyl chain, one can assume that the base peak at 20 V is a product ion (that includes only the N-alkyl chain) produced from fragmentation at either the N–C4 or αC-βC bond. Therefore, the data in this experiment reveal that fentanyl analogs readily produce product ions incorporating the piperidine ring and N-alkyl chain but rarely produce the acyl group. While a protonated molecular ion is an extremely valuable piece of information to possess, so are MSMS data regarding the acyl group. Furthermore, positive APCI mass spectra for fentanyl structures were compared to electron ionization (EI) mass spectra from traditionally implemented GC-MS analysis. APCI readily produces protonated molecular ions while such molecular structure is typically not observed in EI. Furthermore, in EI-GC-MS, fentanyl analogs have a preference for fragmentation at the CO-N amide bond, producing product ion E. Structure E in EI spectra for fentanyls are typically the base peaks. Because of the difference in voltages and how the two methods indirectly or directly ionize the molecule, the base and molecular ion peaks appear to be vastly different. Compared to APCI, EI makes identification of the molecular ion complex without a spectral library and does not behave in a predictable pattern as in APCI. In APCI, the obtained major fragments are useful to distinguish between different positional isomers. APCI and EI both produce broad spectra and can be combined for easier identification of fentanyl analogs since APCI often provides information on the N–C4 bond and the N-αC bond, while EI can more readily provide information on the amide chain as either the base peak or one of the majorly observed peaks. The effective combined use of the two different ionization methods provides information about different sites on a fentanyl molecule and is useful for gathering structural information.

## Supporting Information Available

Tabulated MSMS data for 74 fentanyl analogues, gas chromatography retention time (rt) and relative retention time (rrt) values for the fentanyl analogues, fentanyl analogues that fragment via pathway A, fentanyl analogs that fragment via pathway B and/or C, fentanyl analogs that fragment via pathway D, depictions of fentanyl analogs for pathways A, B/C, and D, pie chart of the distribution of the fentanyl analog fragmentation sites, selected analogs that produce product ions B, C, and E, bar graphs of fragmentation pathways observed at different voltages grouped by increasing mass, comparison of fragmentation pathways produced by EI and APCI (PDF)

## References

[ref1] Chambers S. A.; Desousa J. M.; Huseman E. D.; Townsend S. D. The DARK Side of Total Synthesis: Strategies and Tactics in Psychoactive Drug Production. ACS Chem. Neurosci. 2018, 9, 2307–2330. 10.1021/acschemneuro.7b00528. 29342356 PMC6205722

[ref2] Stanley T. H. The Fentanyl Story. J. of Pain 2014, 15 (12), 1215–1226. 10.1016/j.jpain.2014.08.010. 25441689

[ref3] Burns S. M.; Cunningham C. W.; Mercer S. L. DARK Classics in Chemical Neuroscience: Fentanyl. ACS Chem. Neurosci. 2018, 9 (10), 2428–2437. 10.1021/acschemneuro.8b00174. 29894151

[ref4] Jannetto P. J.; Helander A.; Garg U.; Janis G. C.; Goldberger B.; Ketha H. The Fentanyl Epidemic and Evolution of Fentanyl Analogs in the United States and the European Union. Clinical Chem. 2019, 65 (2), 242–253. 10.1373/clinchem.2017.281626. 30305277

[ref5] Ciccarone D. The Triple Wave Epidemic: Supply and Demand Drivers of the US Opioid Overdose Crisis. International J. of Drug Policy 2019, 71, 183–188. 10.1016/j.drugpo.2019.01.010. PMC6675668 30718120

[ref6] Moore C.; Marinetti L.; Coulter C.; Crompton K. Analysis of Pain Management Drugs, Specifically Fentanyl, in Hair: Application to Forensic Specimens. Forensic Sci. Int. 2008, 176 (1), 47–50. 10.1016/j.forsciint.2007.06.027. 18006260

[ref7] Montanari S.; Davani L.; Terenzi C.; Maltoni M.; Andrisano V.; De Simone A.; Ricci M. Fentanyl Pharmacokinetics in Blood of Cancer Patients by Gas Chromatography – Mass Spectrometry. J. Pharm. Biomed. Anal. 2022, 219, 114913 10.1016/j.jpba.2022.114913. 35810723

[ref8] Romero A.; Miranda H. F.; Puig M. M. Antinociceptive Effects of Morphine, Fentanyl, Tramadol and their Combination, in Morphine-tolerant Mice. Pharmacol., Biochem. Behav. 2010, 97 (2), 363–369. 10.1016/j.pbb.2010.09.005. 20843476

[ref9] Claxton A. R.; McGuire G.; Chung F.; Cruise C. Evaluation of Morphine Versus Fentanyl for Postoperative Analgesia After Ambulatory Surgical Procedures. Anesth. Analg. 1997, 84, 509–514. 10.1097/00000539-199703000-00008. 9052292

[ref10] Dudley S.; Bonelloh D.; Lopez-Aranda J.; Moreno M.; Clavel T.; Kjelstad B.; Restrepo J. J. Mexico’s Role in the Deadly Rise of Fentanyl; Wilson Cent. Woodrow Wilson International Center for Scholars-The Mexico Institute. 2019.

[ref11] Tennyson K. M.; Ray C. S.; Maass K. T. Fentanyl and Fentanyl Analogues: Federal Trends and Trafficking Patterns. United States Sentencing Commission 2021.

[ref12] Barrios R.; Rosen W. L.; Lawrence S. V. Illicit Fentanyl and China’s Role. Congressional Research Service. 2021. URL:https://crsreports.congress.gov/product/pdf/IF/IF10890.

[ref13] Davidson J. T.; Sasiene Z. J.; Jackson G. P. The Influence of Chemical Modifications on the Fragmentation Behavior of Fentanyl and Fentanyl-Related Compounds in Electrospray Ionization Tandem Mass Spectrometry. Drug Test. Anal. 2020, 12 (7), 957–967. 10.1002/dta.2794. 32246896

[ref14] Gupta P. K.; Ganesan K.; Pande A.; Malhotra R. C. A Convenient One Pot Synthesis of Fentanyl. J. Chem. Res. 2005, 2005, 452–453. 10.3184/030823405774309078.

[ref15] Fiorentin T. R.; Krotulski A. J.; Martin D. M.; Browne T.; Triplett J.; Conti T.; Logan B. K. Detection of Cutting Agents in Drug-Positive Seized Exhibits within the United States. J. Forensic Sci. 2019, 64 (3), 888–896. 10.1111/1556-4029.13968. 30485426

[ref16] Canfield J. R.; Agarwal S.; Fortener S. K.; Sprague J. E. Fentanyl Detection Using Eosin Y Paper Assays. J. Forensic Sci. 2020, 65 (5), 1432–1442. 10.1111/1556-4029.14437. 32347988

[ref17] Lockwood T. E.; Vervoordt A.; Lieberman M. High Concentrations of Illicit Stimulants and Cutting Agents cause False Positives on Fentanyl Test Strips. Harm Reduction J. 2021, 18 (1), 1–9. 10.1186/s12954-021-00478-4. PMC7941948 33750405

[ref18] Nunez J.; DeJoseph M. E.; Gill J. R. Xylazine, a Veterinary Tranquilizer, Detected in 42 Accidental Fentanyl Intoxication Deaths. Am. J. Forensic Med. Pathol. 2021, 42 (1), 9–11. 10.1097/PAF.0000000000000622. 33031124

[ref19] Frank R. G.; Pollack H. A. Addressing the Fentanyl Threat to Public Health. New England J. Med. 2017, 376 (7), 605–607. 10.1056/NEJMp1615145. 28199808

[ref20] Mattson C. L.; Tanz L. J.; Quinn K.; Kariisa M.; Patel P.; Davis N. L. Trends and Geographic Patterns in Drug and Synthetic Opioid Overdose Deaths -United States, 2013–2019. Centers for Disease Control and Prevention (CDC) 2021, 70 (6), 202–207. 10.15585/mmwr.mm7006a4. PMC7877587 33571180

[ref21] Kuhlman J. J. Jr.; McCaulley R.; Valouch T. J.; Behonick G. S. Fentanyl Use, Misuse, and Abuse: A Summary of 23 Postmortem Cases. J. Anal. Toxicol. 2003, 27 (7), 499–504. 10.1093/jat/27.7.499. 14607006

[ref22] Vo Q. N.; Mahinthichaichan P.; Shen J.; Ellis C. R. How μ-Opioid Receptor Recognizes Fentanyl. Nat. Commun. 2021, 12, 984 10.1038/s41467-021-21262-9. 33579956 PMC7881245

[ref23] Armenian P.; Vo K. T.; Barr-Walker J.; Lynch K. L. Fentanyl, Fentanyl Analogs and Novel Synthetic Opioids: A Comprehensive Review. Neuropharmacology 2018, 134, 121–132. 10.1016/j.neuropharm.2017.10.016. 29042317

[ref24] Wilson N.; Kariisa M.; Seth P.; Smith H.; Davis N. L. Drug and Opioid-Involved Overdose Deaths — United States, 2017–2018. MMWR Morb. Mortal. Wkly. Rep. 2020, 69, 290–297. 10.15585/mmwr.mm6911a4. 32191688 PMC7739981

[ref25] Spencer M. R.; Garnett M. F.; Miniño A. M. Drug Overdose Deaths in the United States, 2002–2022. NCHS Data Brief, no. 491 2024, 10.15620/cdc:135849. 36598401

[ref26] Oliva J. D.; El-Sabawi T. The ″ New″ Drug War. Va. Law Rev. 2024, 110, 1103.

[ref27] Garg A.; Solas D. W.; Takahashi L. H.; Cassella J. V. Forced Degradation of Fentanyl: Identification and Analysis of Impurities and Degradants. J. Pharm. Biomed. Anal. 2010, 53 (3), 325–334. 10.1016/j.jpba.2010.04.004. 20462721

[ref28] Denis E. H.; Bade J. L.; Renslow R. S.; Morrison K. A.; Nims M. K.; Govind N.; Ewing R. G. Proton Affinity Values of Fentanyl and Fentanyl Analogues Pertinent to Ambient Ionization and Detection. J. Am. Soc. Mass Spectrom. 2022, 33 (3), 482–490. 10.1021/jasms.1c00320. 35041405

[ref29] Stauffer E.; Dolan J. A.; Newman R. Gas Chromatography and Gas Chromatography—Mass Spectrometry; Academic Press: Burlington, 2008; pp 235–293. 10.1016/B978-012663971-1.50012-9.

[ref30] Dueñas M. E.; Peltier-Heap R. E.; Leveridge M.; Annan R. S.; Büttner F. H.; Trost M. Advances in High-throughput Mass Spectrometry in Drug Discovery. EMBO Mol. Med. 2023, 15, e14850 10.15252/emmm.202114850. 36515561 PMC9832828

[ref31] Haglund P.; Harju M.; Danielsson C.; Marriott P. Effects of Temperature and Flow Regulated Carbon Dioxide Cooling in Longitudinally Modulated Cryogenic Systems for Comprehensive Two-dimensional Gas Chromatography. J. Chromatogr. A 2002, 962 (1), 127–134. 10.1016/S0021-9673(02)00433-8. 12198957

[ref32] Deport C.; Ratel J.; Berdagué J.-L.; Engel E. Comprehensive Combinatory Standard Correction: A Calibration Method for Handling Instrumental Drifts of Gas Chromatography–Mass Spectrometry Systems. J. Chromatogr. A 2006, 1116 (1), 248–258. 10.1016/j.chroma.2006.03.092. 16631179

[ref33] Trinklein T. J.; Schöneich S.; Sudol P. E.; Warren C. G.; Gough D. V.; Synovec R. E. Total-transfer Comprehensive Three-dimensional Gas Chromatography with Time-of-flight Mass Spectrometry. J. Chromatogr. A 2020, 1634, 461654 10.1016/j.chroma.2020.461654. 33166893

[ref34] Yang S.; Brereton S. M.; Wheeler M. D.; Ellis A. M. Soft or Hard Ionization of Molecules in Helium Nanodroplets? An Electron Impact Investigation of Alcohols and Ethers. Phys. Chem. Chem. Phys. 2005, 7 (24), 4082–4088. 10.1039/b511628g. 16474872

[ref35] Schepler C.; Sklorz M.; Passig J.; Famiglini G.; Cappiello A.; Zimmermann R. Flow Injection of Liquid Samples to a Mass Spectrometer with Ionization under Vacuum Conditions: A Combined Ion Source for Single-Photon and Electron Impact Ionization. Anal. Bioanal. Chem. 2013, 405 (22), 6953–6957. 10.1007/s00216-013-7151-3. 23812882

[ref36] Eschner M. S.; Gröger T. M.; Horvath T.; Gonin M.; Zimmermann R. Quasi-Simultaneous Acquisition of Hard Electron Ionization and Soft Single-Photon Ionization Mass Spectra during GC/MS Analysis by Rapid Switching between Both Ionization Methods: Analytical Concept, Setup, and Application on Diesel Fuel. Anal. Chem. 2011, 83 (10), 3865–3872. 10.1021/ac200356t. 21500772

[ref37] Amigo J. M.; Skov T.; Coello J.; Maspoch S.; Bro R. Solving GC-MS Problems with PARAFAC2. TrAC Trends in Anal. Chem. 2008, 27 (8), 714–725. 10.1016/j.trac.2008.05.011.

[ref38] Héberger K.; Kowalska T. Thermodynamic Significance of Boiling Point Correlations for Alkylbenzenes in Gas Chromatography: Extension of Trouton’s Rule. J. Chromatogr. A 1999, 845 (1), 13–20. 10.1016/S0021-9673(99)00289-7.

[ref39] Luong J.; Gras R.; Mustacich R.; Cortes H. Low Thermal Mass Gas Chromatography: Principles and Applications. J. Chromatogr. Sci. 2006, 44 (5), 253–261. 10.1093/chromsci/44.5.253. 16774710

[ref40] Vincelet C.; Roussel J. M.; Benanou D. Experimental Designs Dedicated to the Evaluation of a Membrane Extraction Method: Membrane-assisted Solvent Extraction for Compounds having Different Polarities by Means of Gas Chromatography–mass Detection. Anal. Bioanal. Chem. 2010, 396 (6), 2285–2292. 10.1007/s00216-009-3449-6. 20127320

[ref41] Bräkling S.; Kroll K.; Stoermer C.; Rohner U.; Gonin M.; Benter T.; Kersten H.; Klee S. Parallel Operation of Electron Ionization and Chemical Ionization for GC–MS Using a Single TOF Mass Analyzer. Anal. Chem. 2022, 94 (15), 6057–6064. 10.1021/acs.analchem.2c00933. 35388701

[ref42] Andrade F. J.; Shelley J. T.; Wetzel W. C.; Webb M. R.; Gamez G.; Ray S. J.; Hieftje G. M. Atmospheric Pressure Chemical Ionization Source. 2. Desorption–Ionization for the Direct Analysis of Solid Compounds. Anal. Chem. 2008, 80 (8), 2654–2663. 10.1021/ac800210s. 18345694

[ref43] Xu L.; Coggon M. M.; Stockwell C. E.; Gilman J. B.; Robinson M. A.; Breitenlechner M.; Lamplugh A.; Crounse J. D.; Wennberg P. O.; Neuman J. A.; Novak G. A.; Veres P. R.; Brown S. S.; Warneke C. Chemical Ionization Mass Spectrometry Utilizing Ammonium Ions (NH_4_^+^ CIMS) for Measurements of Organic Compounds in the Atmosphere. Atmos. Meas. Technol. 2022, 15 (24), 7353–7373. 10.5194/amt-15-7353-2022.

[ref44] Du B.; Shen M.; Pan Z.; Zhu C.; Luo D.; Zeng L. Trace Analysis of Multiple Synthetic Phenolic Antioxidants in Foods by Liquid Chromatography–tandem Mass Spectrometry with Complementary Use of Electrospray Ionization and Atmospheric Pressure Chemical Ionization. Food Chem. 2022, 375, 131663 10.1016/j.foodchem.2021.131663. 34848092

[ref45] de Koster C. G.; Schoenmakers P. J. Chapter 3.1 - History of liquid chromatography—mass spectrometry couplings. Hyphenations of Capillary Chromatography with Mass Spectrometry 2020, 279–295. 10.1016/B978-0-12-809638-3.00007-7.

[ref46] Stokes P.; Parker D.; Mosely J. Modification of a Gas Chromatography/atmospheric Pressure Chemical Ionisation Time-of-flight Mass Spectrometer as an Alternative to Automated Atmospheric Pressure Solids Analysis Probe. Rapid Commun. Mass Spectrom. 2014, 28 (18), 2024–2030. 10.1002/rcm.6992. 25132303

[ref47] Gómez-Pérez M. L.; Plaza-Bolaños P.; Romero-González R.; Vidal J. L.M.; Frenich A. G. Evaluation of the Potential of GC-APCI-MS for the Analysis of Pesticide Residues in Fatty Matrices. J. Am. Soc. Mass Spectrom. 2014, 25 (5), 899–902. 10.1007/s13361-014-0849-4. 24658807

[ref48] Gottardo R.; Sorio D.; Ballotari M.; Tagliaro F. First Application of Atmospheric-pressure Chemical Ionization Gas Chromatography Tandem Mass Spectrometry to the Determination of Cannabinoids in Serum. J. Chromatogr. A 2019, 1591, 147–154. 10.1016/j.chroma.2019.01.041. 30679047

[ref49] Singh G.; Gutierrez A.; Xu K.; Blair I. A. Liquid Chromatography/Electron Capture Atmospheric Pressure Chemical Ionization/Mass Spectrometry: Analysis of Pentafluorobenzyl Derivatives of Biomolecules and Drugs in the Attomole Range. Anal. Chem. 2000, 72 (14), 3007–3013. 10.1021/ac000374a. 10939360

[ref50] Dams R.; Murphy C. M.; Choo R. E.; Lambert W. E.; De Leenheer A. P.; Huestis M. A. LC–Atmospheric Pressure Chemical Ionization-MS/MS Analysis of Multiple Illicit Drugs, Methadone, and Their Metabolites in Oral Fluid Following Protein Precipitation. Anal. Chem. 2003, 75 (4), 798–804. 10.1021/ac026111t. 12622369

[ref51] Bogusz M. J.; Maier R.-D.; Krüger K.-D.; Kohls U. Determination of Common Drugs of Abuse in Body Fluids Using One Isolation Procedure and Liquid Chromatography-Atmospheric-Pressure Chemical-Ionization Mass Spectrometry*. J. Anal. Toxicol. 1998, 22 (7), 549–558. 10.1093/jat/22.7.549. 9847004

[ref52] Knoke L.; Rettberg N. Evaluation and Optimization of APGC Parameters for the Analysis of Selected Hop Essential Oil Volatiles. ACS Omega 2021, 6 (44), 29932–29939. 10.1021/acsomega.1c04426. 34778665 PMC8582035

[ref53] Majrashi M.; Almaghrabi M.; Fadan M.; Fujihashi A.; Lee W.; Deruiter J.; Clark C. R.; Dhanasekaran M. Dopaminergic Neurotoxic Effects of 3-TFMPP Derivatives. Life Sci. 2018, 209, 357–369. 10.1016/j.lfs.2018.07.052. 30067941

[ref54] Jalali R.; Dmochowska P.; Godlewska I.; Balmas J.; Młynarska K.; Narkun K.; Zawadzki A.; Wojnar M. Designers Drugs-A New Challenge to Emergency Departments-An Observational Study in Poland. Medicina 2020, 56 (7), 354 10.3390/medicina56070354. 32708850 PMC7404717

[ref55] Goodwin S. para-Bromofentanyl GCMS EI Spectra. Cayman Chemical 2019, 1. URL: https://cdn.caymanchem.com/cdn/gcms/27536-0560956-GCMS.pdf, Oct 9, 2024.

[ref56] Jianmei L. 3′-methyl Acetyl fentanyl GCMS EI Spectra. Cayman Chemical 2018, 1. URL: https://cdn.caymanchem.com/cdn/gcms/25474-0542349-GCMS.pdf, Oct 9, 2024.

[ref57] Jianmei L. N-Methylnorfentanyl GCMS EI Spectra. Cayman Chemical 2018, 1. URL: https://cdn.caymanchem.com/cdn/gcms/24446-0524407-GCMS.pdf, Oct 9, 2024.

[ref58] Goodwin S. N-(DOI) Fentanyl GCMS EI Spectra. Cayman Chemical 2020, 1. URL: https://cdn.caymanchem.com/cdn/gcms/28990-0576093-GCMS.pdf, Oct 9, 2024.

