# Supplementary material for: Analysis of Fentanyl and Fentanyl Analogs Using Atmospheric Pressure Chemical Ionization Gas Chromatography–Mass Spectrometry (APCI-GC-MS)
Source: J Am Soc Mass Spectrom. 2025 Feb 3;36(3):587–600. doi: 10.1021/jasms.4c00455 (PMC11887434; doi:10.1021/jasms.4c00455)
Supplement: Supplementary file 1 — js4c00455_si_001.pdf [file js4c00455_si_001.pdf]

Supporting Information

Analysis of Fentanyl and Fentanyl Analogs using Atmospheric Pressure Chemical Ionization Gas Chromatography-Mass Spectrometry (APCI-GC-MS)

Karen A. Reyes Monroy, Richard McCrary, Isabelle Parry, Catherine Webber, Teresa D. Golden, Guido F. Verbeck*

1155 Union Circle #30570 University of North Texas, Department of Chemistry, Denton, TX 76203

* to whom correspondence should be addressed

Table of Contents

Table S1. Tabulated MSMS data for 74 fentanyl analogues. Precursor ions contain an (*), base peaks are bolded, superscript ($\#^{A}$) indicates the m/z value was obtained from cleavage at $C\text{-}N^{4}$, ($\#^{B}$) cleavage at $C\text{-}N^{\alpha}$, ($\#^{C}$) cleavage at $C^{\alpha}\text{-}C^{\beta}$, ($\#^{D}$) cleavage at piperidine ring, and ($\#^{E}$) cleavage at CO-N amide bond structure..,…………………………………………….…...………3-16

Table S2. Gas chromatography retention time (rt) and relative retention time (rrt) values for the fentanyl analogues relative a caffeine standard………………………………………………17-19

Table S3. Fentanyl analogues that fragment via pathway A, resulting in the abundant formation of product ion A………………………………………………………………………...…..20-21

Table S4. Fentanyl analogs that fragment via pathway B and/or C, thus predominantly generating ions B and/or C…………………………….……………………………………...21

Table S5. Fentanyl analogs that fragment via pathway D, thus predominantly fragmenting at the piperidine ring, and resulting in the formation of product ion D………………………………...22

Figure S1. Depiction of 15 analogs that fragment primarily via pathway A…………….…..…….23

Figure S2. Depiction of another 15 analogs that fragment primarily via pathway A…………...24

Figure S3. Depiction of the last 10 analogs that fragment primarily via pathway A…………....25

Figure S4. Depiction of 15 analogs that fragment primarily via pathway B/C………….....…….26

Figure S5. Depiction of the last 14 analogs that fragment primarily via pathway B/C……..…..27

Figure S6. Depiction of 4 analogs that primarily follow pathway D…………………….....…….28

Figure S7. Pie chart depicting the distribution of primary fragmentation sites among the 74 fentanyl analogs analyzed. Of the fentanyl analogs studied, 54% primarily fragmented at site A (the amide N-C4 bond), while 40% fragmented at sites B and/or C (the n-alkyl chain). Only 5% exhibited fragmentation primarily at site D (the piperidine ring)……………………………….28

Figure S8. Four of the eleven analogs that produce solely product ion B at 10-50 V. These analogs generally contain a methyl at the alpha carbon of the alkyl chain and three functional groups at the phenyl moiety, two of which are methoxy groups…………………………..….29

Figure S9. Four of the ten fentanyl analogs that produced product ion C at 20- 50V. These specific compounds contain highly stabilized and substituted R4 groups that drive fragmentation to occur primarily at site C.……………………………………………………………...….29

Figure S10. Illustration of pathway E depicting the structures of norsufentanil, senecioylfentanyl, tigloylfentanyl, para-toluoylfentanyl, para-chloro-furanylfentanyl 3-furancarboxamide, 2,3-benzodioxolefentanyl, and para-methyl cyclopentyl fentanyl with corresponding functional group R1 highlighted in green……………………………………..30

Figure S11. Fragmentation data at different voltages for the 250-300 m/z compounds……………………………………………………………………………………30

Figure S12. Fragmentation data at different voltages for the 301-350 m/z compounds……………………………………………………………………………………31

Figure S13. Fragmentation data at different voltages for the 351-400 m/z compounds……………………………………………………………………………………31

Figure S14. Fragmentation data at different voltages for the 401-450 m/z compounds……………………………………………………………………………………32

Figure S15. Fragmentation data at different voltages for the 451-500 m/z compounds……………………………………………………………………………………32

Figure S16. Fragmentation data at different voltages for the 501-550 m/z compounds……………………………………………………………………………………33

Figure S17. Illustration of APCI data (left) from our study and EI data (right) for para-bromofentanyl. (EI data used with permission from Cayman Chemical).………………………33

Table S1. Tabulated MSMS data for 74 fentanyl analogues run at collision energies of 10, 20, 30, 40, and 50 V and indicating product ions. Precursor ions contain an (*), base peaks are bolded, superscript (#^{A}) indicates the m/z value was obtained from cleavage at C-N^{4}, (#^{B}) cleavage at C-N^{α}, (#^{C}) cleavage at C^{α}-C^{β}, (#^{D}) cleavage at piperidine ring, and (#^{E}) cleavage at CO-N amide bond structure. (The masses are listed with their relative intensities in parenthesis).

**Pathway A**			
**Fentanyl Analogue**	**Chemical Formula**	**Mass (Relative Intensity)**	**Expected Precursor [M+H]+**
**Norsufentanil**	**C16H24N2O2**	**276.18**	**277.18**
	**CID V**	**Dissociation Pathways Observed**	**Detected Ions**
	**10**	**277*(100)**	Precursor
	**20**	**147^{A}(100),** 57^{E1}(92.49)	A, E1
	**30**	**57^{E}(100),** 147^{A}(37.02)	A, E1
	**40**	127^{A}(45.87→**57^{E}(100)**	A, E1
	**50**	**127^{A}(100)**	A, E1
**Despropionyl meta-Methylfentanyl**	**C20H26N2**	**294.21**	**295.21**
	**CID V**	**Dissociation Pathways Observed**	**Detected Ions**
	**10**	**295*(100)**→ 188^{A}(36)	Precursor, A
	**20**	**188^{A}(100)**	A
	**30**	188^{A}(26)→**105^{B}(100)**	A, B
	**40**	**105^{B}(100)**	B
	**50**	**105^{B}(100)**→79(30) →77(21)	B
**Despropionyl para-Fluorofentanyl**	**C19H23FN2**	**298.18**	**299.18**
	**CID V**	**Dissociation Pathways Observed**	**Detected Ions**
	**10**	**299*(100)**	Precursor,
	**20**	**188^{A}(100)**	A
	**30**	188^{A}(30)→**105^{B}(100)**	A, B
	**40**	**105^{B}(100)**	B
	**50**	**$105^{B(}$100)**→79(34)	B
**Despropionyl 2'-fluoro ortho-Fluorofentanyl**	**C19H22F2N2**	**316.18**	**317.18**
	**CID V**	**Dissociation Pathways Observed**	**Detected Ions**
	**10**	**317*(100)→206^{A}(32)**	Precursor
	**20**	**206^{A}(100)**	A
	**30**	206^{A}(65)→**123^{B}(100)**	A, B
	**40**	**123^{B}(100)**→103(80)	B
	**50**	123^{B}(35)→**103(100)**→77(33)	B
**Acetyl fentanyl**	**C21H26N2O**	**322.20**	**323.20**
	**CID V**	**Dissociation Pathways Observed**	**Detected Ions**
	**10**	**323*(100)**	Precursor
	**20**	323*(53)→**188^{A}(100)**	Precursor, A

	**30**	188^{A}(69)→**105^{B}(100)**	A, B
	**40**	**105^{B}(100)**	B
	**50**	**105^{B}(100)**	B
**2'-methyl Acetyl fentanyl**	**C22H28N2O**	**336.22**	**337.22**
	**CID V**	**Dissociation Pathways Observed**	**Detected Ions**
	**10**	**337^{*}(100)**	Precursor
	**20**	**337^{*}(53)→202^{A}(100)**	Precursor, A
	**30**	**202^{A}(40)→119^{B}(100)**	A, B
	**40**	**119^{B}(100)**	B
	**50**	**119^{B}(100)**	B
**3'-methyl Acetyl fentanyl**	**C22H28N2O**	**336.22**	**337.22**
	**CID V**	**Dissociation Pathways Observed**	**Detected Ions**
	**10**	**337^{*}(100)**	Precursor
	**20**	337^{*}(54)→**202^{A}(100)**	Precursor, A
	**30**	202^{A}(40)→**119^{B}(100)**	A, B
	**40**	**119^{B}(100)**	B
	**50**	**119^{B}(33)**→117(43)→91(56)	B
**meta-methyl Acetyl fentanyl**	**C22H28N2O**	**336.22**	**337.22**
	**CID V**	**Dissociation Pathways Observed**	**Detected Ions**
	**10**	**337^{*}(100)**	Precursor
	**20**	337(73)→**188^{A}(100)**	Precursor, A
	**30**	188^{A}(66)→**105^{B}(100)**	A, B
	**40**	**105^{B}(100)**	B
	**50**	**105^{B}(100)**	B
**(±)-cis-Isofentanyl**	**C22H28N2O**	**336.22**	**336.22**
	**CID V**	**Dissociation Pathways Observed**	**Detected Ions**
	**10**	**335^{*}(100)**	Precursor
	**20**	335^{*}(76), **186^{A}(100)**→160(89)→120(69)	Precursor, A
	**30**	160(47)→120(46)→118(53)→**91^{B}(100)**	A, B
	**40**	**91^{B}(100**	B
	**50**	**91^{B}(100)**	B
**Fentanyl Methyl Carbamate**	**C21H26N2O2**	**338.20**	**339.20**
	**CID V**	**Dissociation Pathways Observed**	**Detected Ions**
	**10**	**339^{*}(100)**	Precursor
	**20**	**188^{A}(100)**	Precursor, A
	**30**	188^{A}(32)→**105^{B}(100)**	A, B
	**40**	**105^{B}(100)**	B
	**50**	**105^{B}(100)**	B
**2'-methyl Fentanyl**	**C23H30N2O**	**350.24**	**351.24**
	**CID V**	**Dissociation Pathways Observed**	**Detected Ions**

	**10**	**351*(100)**	Precursor
	**20**	**351*(56)→202^{A}(100)**	Precursor, A
	**30**	**119**^{B}(100)	A, B
	**40**	**119**^{B}(100)	B
	**50**	**119**^{B}(100)→117(39)→91(59)	B
**3'-methyl Fentanyl**	**C23H30N2O**	**350.24**	**351.24**
	**CID V**	**Dissociation Pathways Observed**	**Detected Ions**
	**10**	**351*(100)**	Precursor
	**20**	351*(76)→**202^{A}(100)**	Precursor, A
	**30**	202^{A}(87)→**119^{B}(100)**	A, B
	**40**	**119^{B}(100)**	B
	**50**	**119^{B}(100)**→117(39)→91(53)	B
**4-methyl Fentanyl**	**C23H30N2O**	**350.24**	**351.24**
	**CID V**	**Dissociation Pathways Observed**	**Detected Ions**
	**10**	**351*(100)**→202^{A}(20)	Precursor, A
	**20**	351*(20)→**202^{A}(100)**	Precursor, A
	**30**	202^{A}(75)→**105^{B}**	A, B
	**40**	202^{A}(20)→**105^{B}(100)**	B
	**50**	**105^{B}(100)**	B
**N,N-Dimethylamido-despropionyl fentanyl**	**C22H29N3O**	**351.23**	**352.23**
	**CID V**	**Dissociation Pathways Observed**	**Detected Ions**
	**10**	**352*(100)**	Precursor
	**20**	352*(38)→**188^{A}(100)**	Precursor, A
	**30**	188^{A}(61)→**105^{B}(100)**	A, B
	**40**	**105^{B}(100)**	B
	**50**	**105^{B}(100)**	B
**meta-fluoro Acrylfentanyl**	**C22H25FN2O**	**352.20**	**353.20**
	**CID V**	**Dissociation Pathways Observed**	**Detected Ions**
	**10**	**353*(100)**	Precursor
	**20**	**188^{A}(100)**	A
	**30**	188^{A}(89)→**105^{B}(100)**	A, B
	**40**	**105^{B}(100)**	B
	**50**	**105^{B}(100)**	B
**b-hydroxy fentanyl**	**C22H28N2O2**	**352.22**	**353.22**
	**CID V**	**Dissociation Pathways Observed**	**Detected Ions**
	**10**	**353*(100)**	Precursor
	**20**	353*(52)→335(82)→**204^{A}(100)**	Precursor, A
	**30**	145(80)→**132(100)**	A, B
	**40**	**145**^{B}(100)	B
	**50**	**91(100)**	B

**2'-Fluorofentanyl**	**C22H27FN2O**	**354.21**	**355.21**
	**CID V**	**Dissociation Pathways Observed**	**Detected Ions**
	**10**	**355*(100)**	Precursor
	**20**	355*(46)→**206^{A}(100)**	A
	**30**	**206^{A}(100)**→123^{B}(73)	A, B
	**40**	**123^{B}(100)**→103(44)	B
	**50**	123^{B}(76)→**103(100)**	B
**3'-Fluorofentanyl**	**C22H27FN2O**	**354.21**	**355.21**
	**CID V**	**Dissociation Pathways Observed**	**Detected Ions**
	**10**	**355*(100)**	Precursor
	**20**	**206^{A}(100)**	A
	**30**	**206^{A}(100)**→123^{B}(44)	A, B
	**40**	206^{A}(53)→**123^{B}(100)**→103(58)→73(44)→55 (57)	A, B
	**50**	123^{B}(65)→**103(100)**→55(37)	B
**4'-Fluorofentanyl**	**C22H27FN2O**	**355.22**	**356.22**
	**CID V**	**Dissociation Pathways Observed**	**Detected Ions**
	**10**	**355*(100)**	Precursor
	**20**	355*(52)→**206^{A}(100)**	A
	**30**	206^{A}(65)→**123^{B}(100)**	A, B
	**40**	**123^{B}(100)**→103(35)	B
	**50**	123^{B}(69)→**103(100)**	B
**meta-methyl Cyclopropyl fentanyl**	**C24H30N2O**	**362.24**	**363.24**
	**CID V**	**Dissociation Pathways Observed**	**Detected Ions**
	**10**	**363*(100)**	Precursor
	**20**	363(40)→**188^{A}(90)**	Precursor, A
	**30**	**188^{A}(100)**→105^{B}(57)	A, B
	**40**	188^{A}(37)→**105^{B}(100)**	B
	**50**	**105^{B}(100)**	B
**Senecioylfentanyl**	**C24H30N2O**	**362.24**	**363.24**
	**CID V**	**Dissociation Pathways Observed**	**Detected Ions**
	**10**	**363*(100)**	Precursor
	**20**	363*(32)→**188^{A}**(100), 83^{E1}(75)	A, E1
	**30**	188^{A}(54)→105^{B}(37), **83^{E1}(100)**	A, B, E1
	**40**	105(48), **83^{E1}(100)**→55(30)	B, E1
	**50**	105(46), 83^{E1}(45)→**55(100)**	B, E1
**Tigloyl fentanyl**	**C24H30N2O**	**362.24**	**363.24**
	**CID V**	**Dissociation Pathways Observed**	**Detected Ions**
	**10**	**363*(100)**→188^{A}(90)	Precursor, A
	**20**	363*(40)→188^{A} (90),**83^{E1}(100)**	A, E1
	**30**	**188^{A}(100)**→105^{B}(50)→55(55), 83^{E1}(70)	A, B, E1
	**40**	**105^{B}(100)**→55(55), 83^{E1}(35)	B, E1
	**50**	105^{B}(80)→**55(100)**	B
**para-methyl Butyryl fentanyl**	**C24H32N2O**	**365.26**	**366.26**

	CID V	Dissociation Pathways Observed	Detected Ions
	**10**	**365*(100)**	Precursor
	**20**	365*(43)→**188^{A}(100)**	Precursor, A
	**30**	188^{A}(76)→**105^{B}(100)**	A, B
	**40**	**105^{B}(100)**	B
	**50**	**105^{B}(100)**	B
**3'-fluoro ortho-Fluorofentanyl**	**C22H26F2N2O**	**372.20**	**373.20**
	**CID V**	**Dissociation Pathways Observed**	**Detected Ions**
	**10**	**373*(100)**	Precursor
	**20**	373*(46)→**206^{A}(100)**	Precursor, A
	**30**	**206^{A}(100)**→123^{B}(68)	A, B
	**40**	206^{A}(43)→**123^{B}(100)**→102(71)→55(39)	A, B
	**50**	**123^{B}(100)**→102(99)→55(50)	B
**Furanyl fentanyl**	**C24H27N2O2**	**374.21**	**375.21**
	**CID V**	**Dissociation Pathways Observed**	**Detected Ions**
	**10**	**375*(100)**	Precursor
	**20**	375*(34)→**188^{A}(100)**	Precursor, A
	**30**	**188^{A}(100)**→105^{B}(97)	A, B
	**40**	188^{A}(49)→**105^{B}(100)**	A, B
	**50**	**105^{B}(100)**	B
**Hexanoyl fentanyl**	**C25H34N2O**	**378.21**	**379.21**
	**CID V**	**Dissociation Pathways Observed**	**Detected Ions**
	**10**	**379*(100)**	Precursor
	**20**	379*(81)→**188^{A}(100)**	Precursor, A
	**30**	**188^{A}(100)**→105^{B}(99)	A, B
	**40**	188^{A}(23)→**105^{B}(100)**→43(31)	A, B
	**50**	**105^{B}(100)**	B
**ortho-fluoro Valeryl fentanyl**	**C24H31FN2O**	**382.24**	**383.24**
	**CID V**	**Dissociation Pathways Observed**	**Detected Ions**
	**10**	**383*(100)**	Precursor
	**20**	383*(47)→**188^{A}(100)**	Precursor, A
	**30**	**188^{A}(100)**→105^{B}(53)	A, B
	**40**	**105^{B}(100)**	B
	**50**	**105^{B}(100)**	B
**para-methoxy Methoxyacetyl fentanyl**	**C23H30N2O3**	**383.23**	**384.23**
	**CID V**	**Dissociation Pathways Observed**	**Detected Ions**
	**10**	**383*(100)**	Precursor
	**20**	**188^{A}(100)**	A
	**30**	188^{A}(70)→**105^{B}(100)**	A, B

	**40**	188^{A}(51)→**105^{B}(100)**	A, B
	**50**	**105^{B}(100)**	B
**para-methyl Cyclopentyl fentanyl**	**C26H34N2O**	**391.27**	**392.27**
	**CID V**	**Dissociation Pathways Observed**	**Detected Ions**
	**10**	**391*(100)**	Precursor
	**20**	**188^{A}(100)**	A
	**30**	**188^{A}(100)**	A
	**40**	105^{B}(93)<br>**69^{E1}(100)**	B, E1
	**50**	105^{B}(96)<br>69^{E1}(72)→ **55(100)**	B, E1
**meta-fluoro Furanyl fentanyl**	**C24H25FN2O2**	**392.19**	**393.19**
	**CID V**	**Dissociation Pathways Observed**	**Detected Ions**
	**10**	**393*(100)**	Precursor
	**20**	**188^{A}(100)**	A
	**30**	188^{A}(86)→**105^{B}(100)**	A, B
	**40**	**105^{B}(100)**	B
	**50**	**105^{B}(100)**	B
**Heptanoyl fentanyl**	**C26H36N2O**	**392.28**	**393.28**
	**CID V**	**Dissociation Pathways Observed**	**Detected Ions**
	**10**	**393*(100)**	Precursor
	**20**	393*(82)→**188^{A}(100)**	Precursor, A
	**30**	**188^{A}(100)**→105^{B}(39)	A, B
	**40**	**105^{B}(100)**	B
	**50**	**105^{B}(100)**	B
**Tetrahydrothiophene fentanyl**	**C24H30N2OS**	**395.22**	**396.22**
	**CID V**	**Dissociation Pathways Observed**	**Detected Ions**
	**10**	**395*(100)**	Precursor
	**20**	**395*(40)→188^{A}(100)**	Precursor, A
	**30**	**188^{A}(100)**→105^{B}(86)	B
	**40**	188^{A}(43)→**105^{B}(100)**	B
	**50**	**105^{B}(100)**	B
**2',5'-dimethoxy Fentanyl**	**C24H32N2O3**	**396.24**	**397.24**
	**CID V**	**Dissociation Pathways Observed**	**Detected Ions**
	**10**	**397*(100)**	Precursor
	**20**	**397*(100)**→248^{A}(96)	Precursor, A
	**30**	**165^{B}(100)**→150(46)	B
	**40**	165^{B}(70)→**150(100)**	B
	**50**	**150(100)**→105(49)	B
**para-Toluoyl fentanyl**	**C27H30N2O**	**398.24**	**399.24**
	**CID V**	**Dissociation Pathways Observed**	**Detected Ions**

	**10**	**399*(100)**	Precursor, A
	**20**	**188^{A}(100)**<br>119^{E1}(49)	A, E1
	**30**	**188^{A}(100)**→105^{B}(39)<br>**119^{E1}(100)**	A, B, E1
	**40**	105^{B}(72)<br>**119^{E1}(100)**	B, E1
	**50**	**105^{B}(100)**→91(81)<br>119^{E1}(73)	B, E1
**para-chloro Furanyl fentanyl 3-furancarboxamide**	**C24H25ClN2O2**	**408.17**	**409.17**
	**CID V**	**Dissociation Pathways Observed**	**Detected Ions**
	**10**	**409*(100)**	Precursor, A
	**20**	**409*(100)**	Precursor, A, E1
	**30**	409*(53)→**188^{A}(100)**	A, E1
	**40**	188^{A}(100)→105^{B}(79)<br>95^{E1}(37)	A, B, E1
	**50**	**105^{B}(100)**	B
**para-Bromofentanyl**	**C22H27BrN2O**	**414.13**	**415.13**
	**CID V**	**Dissociation Pathways Observed**	**Detected Ions**
	**10**	**415*(100)**	Precursor
	**20**	415*(57)→**188^{A}(100)**	Precursor, A
	**30**	**188^{A}(100)**→105^{B}(45)	A, B
	**40**	**105^{B}(100)**	B
	**50**	**105^{B}(100)**	B
**Phenoxyacetyl fentanyl**	**C27H30N2O2**	**414.24**	**415.24**
	**CID V**	**Dissociation Pathways Observed**	**Detected Ions**
	**10**	**415*(100)**	Precursor
	**20**	415***(57)→188^{A}(100)**	Precursor, A
	**30**	**188^{A}(100)**→105^{B}(65)	A, B
	**40**	188^{A}(37)→**105^{B}(100)**	A, B
	**50**	**105^{B}(100)**	B
**2,3-Benzodioxole fentanyl**	**C27H28N2O3**	**428.22**	**429.22**
	**CID V**	**Dissociation Pathways Observed**	**Detected Ions**
	**10**	**429*(100)**	Precursor
	**20**	149^{E1}(80)<br>**188^{A}(100)**	A, E1
	**30**	**188^{A}(100)**→147(44)→105^{B}(64)→73(80)<br>149 ^{E1}(70)	A, B, E1
	**40**	147(39)→**105^{B}(100)**→73(91)<br>149^{E1}(60)	A, B, E1
	**50**	**105^{B}(100)**→73(94)<br>149^{E1}(57)	B, E1

**N-(2C-TFM) Fentanyl**	**C25H31F3N2O3**	**464.23**	**465.23**
	**CID V**	**Dissociation Pathways Observed**	**Detected Ions**
	**10**	**465*(100)**	Precursor
	**20**	**465*(100)**	Precursor
	**30**	**316^{A}(100)**→233^{B}(72)	A, B
	**40**	316^{A}(37)→**233^{B}(100)**<br>218^{C}(77)	A, B, C
	**50**	**218^{C}(100)**	C
**N-(2C-I) Fentanyl**	**C24H31IN2O3**	**522.14**	**523.14**
	**CID V**	**Dissociation Pathways Observed**	**Detected Ions**
	**10**	**523*(100)**	Precursor
	**20**	**523*(100)**	Precursor
	**30**	**374^{A}(100)**→291^{B}(88)→275(66)→133(57)→98(69)	A, B
	**40**	**291^{B}(41)**→276(54)	B
	**50**	291^{B}(41)→276(54)→**149(100)**→121(63)	B
**Pathway B/C**			
**N-(Phentermine) Fentanyl**	**C24H32N2O**	**364.25**	**365.25**
	**CID V**	**Dissociation Pathways Observed**	**Detected Ions**
	**10**	**365*(100)**	Precursor
	**20**	365*(42)→233^{B}(55)→**84(100)**	Precursor, B
	**30**	**84^{B}(100)**	B
	**40**	91^{B}(63)→**84(100)**	B
	**50**	91^{B}(85)→ **84(100)**→55(96)	B
**N-(3-ethylindole) Norfentanyl**	**C24H29N3O**	**375.23**	**376.23**
	**CID V**	**Dissociation Pathways Observed**	**Detected Ions**
	**10**	376*(100)	Precursor
	**20**	376*(73), **245^{C}(100)**→189(43), 144^{B}(78)	Precursor, B, C
	**30**	189(59), **144^{B}(100)**	B,C
	**40**	**146(100)**, 144^{B}(78)→43(51)	B,C
	**50**	144^{B}(93)→43(71), **117(100)**	B,C
**N-(2-APB) Fentanyl**	**C25H30N2O2**	**390.23**	**391.23**
	**CID V**	**Dissociation Pathways Observed**	**Detected Ions**
	**10**	391*(100)	Precursor
	**20**	391*(32)→242^{A}(37)<br>**259^{C}(100)**	Precursor, A, C
	**30**	146(56)→110(36)<br>**159(100)**	A, C
	**40**	159.24(73)→**131.26(100)**<br>146.21(32)→110.39(28)→58.28(28)	A, C
	**50**	159.24(31)→**131.26(100)**<br>91(32)	A, C
**N-(6-APB) Fentanyl**	**C25H30N2O2**	**390.23**	**391.23**
	**CID V**	**Dissociation Pathways Observed**	**Detected Ions**

	**10**	**391*(100)**	Precursor
	**20**	**391*(100)**→159^{B}(36)	Precursor, B
	**30**	**159^{B}(100)**→131(91)	B
	**40**	**131(100)**	B
	**50**	**131(100)**	B
**N-(6-APDB) Fentanyl**	**C25H32N2O2**	**392.25**	**393.25**
	**CID V**	**Dissociation Pathways Observed**	**Detected Ions**
	**10**	**393*(100)**	Precursor
	**20**	**393*(100)**<br>259^{C}(32)	Precursor, C
	**30**	**259^{C}(32)**<br>**161^{B}(100)**→133(60)→98(36)→84(43)	B, C
	**40**	161^{B}(48)→**133(100)**	B
	**50**	**133(100)**	B
**N-(MDA) Fentanyl**	**C24H30N2O3**	**394.23**	**395.23**
	**CID V**	**Dissociation Pathways Observed**	**Detected Ions**
	**10**	**395*(100)**	Precursor
	**20**	**395*(100)**→245^{A}(31)→163^{B}(48)	Precursor, A, B
	**30**	**163^{B}(100)**→133(43)→105(34)	B
	**40**	163^{B}(86)→135(45)→133(70)→**105(100)**	B
	**50**	133(72)→**105(100)**→79(30)	B
**2',4'-dimethoxy Fentanyl**	**C24H32N2O3**	**396.24**	**397.24**
	**CID V**	**Dissociation Pathways Observed**	**Detected Ions**
	**10**	**397*(100)**	Precursor
	**20**	397*(70)→**165^{B}(100)**	Precursor, B
	**30**	**165^{B}(100)**	B
	**40**	**165^{B}(100)**	B
	**50**	165^{B}(97) →**105(100)**	B
**2',3'-dimethoxy Fentanyl**	**C24H32N2O3**	**396.24**	**397.24**
	**CID V**	**Dissociation Pathways Observed**	**Detected Ions**
	**10**	**397*(100)**	Precursor
	**20**	**397*(100)**→248^{A}(42)→165^{B}(73)	Precursor, A, B
	**30**	**165^{B}(100)**	A, B
	**40**	**165^{B}(100)**→150(72)→105(38)	A, B
	**50**	165^{B}(47)→**150(100)**	B
**2',6'-dimethoxy Fentanyl**	**C24H32N2O3**	**396.24**	**397.24**
	**CID V**	**Dissociation Pathways Observed**	**Detected Ions**
	**10**	**397*(100)**	Precursor
	**20**	**397*(100)**→248^{B}(42)→165^{A}(73)	Precursor, A, B
	**30**	**165^{A}(100)**	A,
	**40**	**165^{A}(100)**→105(34)	A
	**50**	**105(100)**→103(46)→79(41)	A

**3',4'-dimethoxy Fentanyl**	**C24H32N2O3**	**396.24**	**397.24**
	**CID V**	**Dissociation Pathways Observed**	**Detected Ions**
	**10**	**397*(100)**	Precursor
	**20**	**397*(100)→**248(51)	Precursor, A
	**30**	**165^{B}(100)**	B
	**40**	**165^{B}(100)→**150(44)	B
	**50**	165^{B}(99)→150(93)→135(51)→**105(100)→**79(80)	B
**3',5'-dimethoxy Fentanyl**	**C24H32N2O3**	**396.24**	**397.24**
	**CID V**	**Dissociation Pathways Observed**	**Detected Ions**
	**10**	**397.65* (100)**	Precursor
	**20**	245^{C}(91)→ 189(41)→158(40)<br>248^{A}(50)<br>**397.65* (100)**	A, C
	**30**	189^{C}(53)→ 179(32)→ **146(100)**<br>248^{A} (31)→ 84(32)	A, C
	**40**	**146^{C}(100)**	C
	**50**	165^{B}(41)→ 44 (63)<br>**146^{C}(100)→** 105(723)→103(44)	B, C
**N-(2,5-DMA) Fentanyl**	**C25H34N2O3**	**410.26**	**411.26**
	**CID V**	**Dissociation Pathways Observed**	**Detected Ions**
	**10**	**411*(100)**	Precursor
	**20**	**411*(100)**	Precursor
	**30**	179^{B}(20)→**411(100)**	B
	**40**	179^{B}(38)→164(47)→**150(100)**	B
	**50**	164(41)→151(74)→**121(100)→**91(36)	B
**N-(2C-D) Fentanyl**	**C25H34N2O3**	**410.26**	**411.26**
	**CID V**	**Dissociation Pathways Observed**	**Detected Ions**
	**10**	**411*(100)**	Precursor
	**20**	**411*(100)→**179^{B}(30)	Precursor, B
	**30**	**179^{B}(100)→**164(39)	B
	**40**	179^{B}(95)→**164(100)**	B
	**50**	179^{B}(51)→**164(100)→**149(54)→119(37)→117(36)→91(33)	B
**N-(2C-E) Fentanyl**	**C26H36N2O3**	**424.27**	**425.27**
	**CID V**	**Dissociation Pathways Observed**	**Detected Ions**
	**10**	**425*(100)**	Precursor
	**20**	**425*(100)→**276^{A}(31)→193^{B}(32)	Precursor, A, B
	**30**	**193^{B}(100)→**178(38)	B
	**40**	193^{B}(46)→**178(100)**	B
	**50**	193^{B}(64)→178(93)→120(75)→**105(100)→**91(70)	B
**N-(2C-G) Fentanyl**	**C26H36N2O3**	**424.27**	**425.27**

	CID V	Dissociation Pathways Observed	Detected Ions
	**10**	**425*(100)**	Precursor
	**20**	**425*(100)**→276^{A}(31)	Precursor, A
	**30**	193^{B}(36)→**178(100)**	B
	**40**	**178^{B}(100)**	B
	**50**	**178^{B}(100)**→163(48)	B
**N-(2C-C) Fentanyl**	**C24H31ClN2O3**	**430.20**	**431.20**
	**CID V**	**Dissociation Pathways Observed**	**Detected Ions**
	**10**	**431*(100)**	Precursor
	**20**	**431*(100)**→282^{A}(48)	Precursor, A
	**30**	**199^{B}(100)**	B
	**40**	**199^{B}(100)**→184(73)	B
	**50**	199^{B}(52)→**184(100)**→73(35)	B
**N-(2C-iP) Fentanyl**	**C27H38N2O3**	**438.29**	**439.29**
	**CID V**	**Dissociation Pathways Observed**	**Detected Ions**
	**10**	**439*(100)**	Precursor
	**20**	**439*(100)**	Precursor
	**30**	**207^{B}(100)**→193(41)	B
	**40**	**207^{B}(100)**→193(79)	B
	**50**	207^{B}(46)→**193(100)**→177(45)→150(37)→ 120(52)→42(36)	B
**N-(DOET) Fentanyl**	**C27H38N2O3**	**438.29**	**439.29**
	**CID V**	**Dissociation Pathways Observed**	**Detected Ions**
	**10**	**439*(100)**	Precursor
	**20**	**439*(100)**→207^{B}(74)	Precursor, B
	**30**	207^{B}(91)→**179(100)**	B
	**40**	192^{B}(79)→**179(100)**	B
	**50**	192^{B}(92)→179(96)→177(45)→**149(100)**→ 119(85)	B
**N-(2C-P) Fentanyl**	**C27H38N2O3**	**438.29**	**439.29**
	**CID V**	**Dissociation Pathways Observed**	**Detected Ions**
	**10**	**439*(100)**	Precursor
	**20**	**439*(100)**→207^{B}(51)	Precursor, B
	**30**	290^{A}(30)→**207^{B}(100)**→192(38)	A, B
	**40**	**207^{B}(100)**→192(81)	B
	**50**	207^{B}(47)→**192(100)**→163(71)→149(55)→ 135(61)	B
**N-(3,4,5-TMA) Fentanyl**	**C26H36N2O4**	**440.27**	**441.27**
	**CID V**	**Dissociation Pathways Observed**	**Detected Ions**
	**10**	**441*(100)**	Precursor
	**20**	**441(100)**, 259^{C}(80), 209^{B}(63)	Precursor, B, C
	**30**	259^{C}(40), 209^{B}(100)	B, C
	**40**	209^{B}(57)→1789(100)	B, C

		203^{C}(66)→110(69)	
	**50**	178(75)<br>146^{C}(83)→107(86)	B, C
**N-(2C-N) Fentanyl**	**C24H31N3O5**	**441.23**	**442.23**
	**CID V**	**Dissociation Pathways Observed**	**Detected Ions**
	**10**	**442*(100)**	Precursor
	**20**	442*(87)→293^{A}(77)→**245^{B}(100)**→189(35)	Precursor, A, B
	**30**	293^{A}(90)→**210(100)**→189(98)→146(39)→57(46)	A
	**40**	293^{A}(55)→210(94)→**151(100)**→146(88)→95(70)	A
	**50**	151(79)→**146(100)**→57(68)	A
**N-(2C-T) Fentanyl**	**C25H34N2O3S**	**442.23**	**443.23**
	**CID V**	**Dissociation Pathways Observed**	**Detected Ions**
	**10**	**443*(100)**	Precursor
	**20**	**443*(100)**→211^{B}(58)	Precursor, B
	**30**	**211^{B}(100)**	B
	**40**	**211^{B}(100)**→196(64)	B
	**50**	**211^{B}(100)**→196(97)→181(84)→166(74)→134(76)	B
**N-(DOC) Fentanyl**	**C25H33ClN2O3**	**444.22**	**445.22**
	**CID V**	**Dissociation Pathways Observed**	**Detected Ions**
	**10**	**445*(100)**	Precursor
	**20**	**445*(100)**	Precursor
	**30**	**213^{B}(100)**→185(46)	B
	**40**	213^{B}(66)→198(45)→**185(100)**	B
	**50**	198(45)→185(70)	B
**N-(2C-T-2) Fentanyl**	**C26H36N2O3S**	**456.24**	**457.24**
	**CID V**	**Dissociation Pathways Observed**	**Detected Ions**
	**10**	**457*(100)**	Precursor
	**20**	**457*(100)**→308^{A}(50)→225^{B}(65)	Precursor, A, B
	**30**	**225^{B}(100)**	B
	**40**	**225^{B}(100)**→210(38)	B
	**50**	225^{B}(65)→**210(100)**→164(49)→134(59)→119(35)	B
**N-(DOBU) Fentanyl**	**C29H42N2O3**	**466.32**	**467.32**
	**CID V**	**Dissociation Pathways Observed**	**Detected Ions**
	**10**	**467*(100)**	Precursor
	**20**	**467*(100)**→235^{B}(87)	Precursor, B
	**30**	**235^{B}(100)**	B
	**40**	**220^{B} (100)**→207(98)→179(85)	B
	**50**	220^{B} (58)→207(66)→179(65)→177(57)→164(69)→151(73)→149(79)→135(77)→132(65)→**121(100)**→57(82)	B
**N-(2C-T-7) Fentanyl**	**C27H38N2O3S**	**470.26**	**471.26**

	**CID V**	**Dissociation Pathways Observed**	**Detected Ions**
	**10**	**471*(100)**	Precursor
	**20**	**471*(100)**→239^{B}(35)	Precursor, B
	**30**	322^{A}(30)→**239^{B}(100)**	A, B
	**40**	322^{A}(32)→**239^{B}(100)**	A, B
	**50**	182^{B}(84)→164(80)→**134(100)**→124(66)	B
**N-(2C-T-4) Fentanyl**	**C27H38N2O3S**	**470.26**	**471.26**
	**CID V**	**Dissociation Pathways Observed**	**Detected Ions**
	**10**	**471*(100)**	Precursor
	**20**	**471*(100)**	Precursor
	**30**	245^{C}(35),**239^{B}(100)**→197(89)	B, C
	**40**	**197^{B}(100)**	B
	**50**	**197 ^{B}(100)**→119(58)→95(60)→44(60)	B
**N-(2C-B) Fentanyl**	**C24H31BrN2O3**	**474.15**	**475.15**
	**CID V**	**Dissociation Pathways Observed**	**Detected Ions**
	**10**	**475*(100)**	Precursor
	**20**	**475*(100)**→326^{A}(42)	Precursor, A
	**30**	326^{A}(45)→**243^{B}(100)**→231(36)→228(44)→ 132(36)→113(37)	A, B
	**40**	**243^{B}(100)**→228(49)	B
	**50**	243^{B}(97)→**228(100)**→213(36)→164(50)→ 121(45)	B
**N-(DOB) Fentanyl**	**C25H33BrN2O3**	**488.17**	**489.17**
	**CID V**	**Dissociation Pathways Observed**	**Detected Ions**
	**10**	**489*(100)**	Precursor
	**20**	**489*(100)**→257^{B}(54)	Precursor, B
	**30**	**257^{B}(100)**→229(50)→178(39)	B
	**40**	257^{B}(51)→242(44)→229(36)→**178(100)**	B
	**50**	**257^{B}(100)**→178(97)→163(98)→73(81)	B
**N-(DOI) Fentanyl**	**C25H33IN2O3**	**536.15**	**537.15**
	**CID V**	**Dissociation Pathways Observed**	**Detected Ions**
		**537*(100)**	Precursor
		**537*(100)**	Precursor
		537*(55), 388^{A}(38), **305^{B}(100)**→178(86)	A, B
		305^{B}(92)→**290(100)**→277(84)→178(82)	B
		277 ^{B} (60)→178(76)→**163(100)**	B
		**Pathway D**	
**(±)-cis-3-methyl Norfentanyl**	**C15H22N2O**	**246.17**	**247.17**
	**CID V**	**Dissociation Pathways Observed**	**Detected Ions**
	**10**	**245.42*(100)** 202^{D}(47)→172(62)	Precursor, D
	**20**	202^{D}(48)→**160(100)**	D
	**30**	160(96)→145(56)→132(59)→**44(100)**	D
	**40**	160(96)→145(47)→→42(57)→**44(100)**	D

		132(47)→130(47)→91(65)	
	**50**	144(50)→→44(97)→42(91)<br>130(70)→**91(100)**	D
**Furanyl norfentanyl**	**C16H18N2O2**	**270.14**	**271.14**
	**CID V**	**Dissociation Pathways Observed**	**Detected Ions**
	**10**	**271*(100)**<br>228^{D}(31)→203(74)→172(63)	Precursor, D
	**20**	203(42)→**160(100)**	D
	**30**	160(93)→**69(100)**→44(67)	D
	**40**	**69(100)**→44(82)→41(62)	D
	**50**	91(99)→**69(100)**→44(93)→41(84)	D
**Despropionyl ortho-Fluorofentanyl**	**C19H23FN2**	**298.18**	**299.18**
	**CID V**	**Dissociation Pathways Observed**	**Detected Ions**
	**10**	299(63)→**158^{D}(100)**	Precursor, D
	**20**	**111(100)**	D
	**30**	**111(100)**	D
	**40**	**111(100)**	D
	**50**	**111(100)**→83(51)	D
**2,3-seco-Fentanyl**	**C22H30N2O**	**338.24**	**339.24**
	**CID V**	**Dissociation Pathways Observed**	**Detected Ions**
	**10**	**339*(100)**→204(17)	Precursor, D
	**20**	**204^{D}(100)**→120(18)	D
	**30**	204^{D}(23)→**120(100)**	D
	**40**	**120(100)**→57(21)	D
	**50**	**120(100)**→57(15)	D

Table S2. Gas chromatography retention time (rt) and relative retention time (rrt) values for the fentanyl analogues relative a caffeine standard.

Compound Name	Mass	Retention time (rt)	Relative retention time (rrt)
(±)-cis-3-methyl Norfentanyl	246.1732	45.19	2.04
meta-methyl Cyclopropyl fentanyl	362.2354	36.46	1.65
Furanyl norfentanyl (hydrochloride)	270.1368	48.66	2.20
Norsufentanil	276.1838	24.50	1.11
Despropionyl meta-Methylfentanyl	294.2096	41.12	1.86
Despropionyl para-Fluorofentanyl	298.1845	39.01	1.76
Despropionyl ortho-Fluorofentanyl	298.1845	55.67	2.51
Despropionyl 2'-fluoro ortho-Fluorofentanyl	316.1751	37.20	1.68
Acetyl fentanyl (hydrochloride)	322.2045	43.90	1.98
meta-methyl Acetyl fentanyl (hydrochloride)	336.2202	45.20	2.04
3'-methyl Acetyl fentanyl (hydrochloride)	336.2202	45.75	2.07
2'-methyl Acetyl fentanyl (hydrochloride)	336.2202	46.21	2.09
(±)-cis-Isofentanyl (hydrochloride)	336.2202	43.28	1.95
Fentanyl Methyl Carbamate	338.1994	42.94	1.94
2,3-seco-Fentanyl (hydrochloride)	338.2358	39.75	1.79
4-methyl Fentanyl (hydrochloride)	350.2358	45.00	2.03
3'-methyl Fentanyl (hydrochloride)	350.2358	46.86	2.12
2'-methyl Fentanyl (hydrochloride)	350.2358	47.41	2.14
N,N-Dimethylamido-despropionyl fentanyl	351.2311	46.25	2.09
meta-fluoro Acrylfentanyl	352.1951	44.32	2.00
β-hydroxy Fentanyl (hydrochloride)	352.2147	52.71	2.38
2'-Fluorofentanyl (hydrochloride)	354.2107	44.59	2.01
3'-Fluorofentanyl (hydrochloride)	354.2107	44.77	2.02

**4'-fluorofentanyl (hydrochloride)**	**354.2107**	**44.87**	**2.03**
**Tigloyl fentanyl**	**362.2358**	**48.03**	**2.17**
**Senecioylfentanyl**	**362.2358**	**48.08**	**2.17**
**para-methyl butyryl fentanyl (hydrochloride)**	**364.2511**	**48.31**	**2.18**
**N-(Phentermine) Fentanyl (hydrochloride)**	**364.2515**	**47.84**	**2.16**
**3'-fluoro ortho-Fluorofentanyl (hydrochloride)**	**372.2013**	**44.25**	**2.00**
**Furanyl fentanyl (hydrochloride)**	**374.1991**	**51.83**	**2.34**
**N-(3-ethylindole) Norfentanyl**	**375.2311**	**59.31**	**2.68**
**Hexanoyl fentanyl (hydrochloride)**	**378.2667**	**50.50**	**2.28**
**ortho-fluoro Valeryl fentanyl (hydrochloride)**	**382.2420**	**48.11**	**2.17**
**N-(2-APB) Fentanyl**	**390.2307**	**53.63**	**2.42**
**N-(6-APB) Fentanyl**	**390.2307**	**53.69**	**2.42**
**meta-fluoro Furanyl fentanyl (hydrochloride)**	**392.1900**	**50.50**	**2.28**
**N-(6-APDB) Fentanyl**	**392.2464**	**55.37**	**2.50**
**Heptanoyl fentanyl (hydrochloride)**	**392.2828**	**52.50**	**2.37**
**Tetrahydrothiophene fentanyl**	**394.2075**	**56.22**	**2.54**
**N-(MDA) Fentanyl (hydrochloride)**	**394.2256**	**53.73**	**2.43**
**2',3'-dimethoxy Fentanyl (hydrochloride)**	**396.2413**	**52.28**	**2.36**
**2',6'-dimethoxy Fentanyl (hydrochloride)**	**396.2413**	**52.68**	**2.38**
**2',5'-dimethoxy Fentanyl (hydrochloride)**	**396.2413**	**53.42**	**2.41**
**3',4'-dimethoxy Fentanyl (hydrochloride)**	**396.2413**	**53.91**	**2.43**
**2',4'-dimethoxy Fentanyl (hydrochloride)**	**396.2413**	**53.94**	**2.44**
**3',5'-dimethoxy Fentanyl (hydrochloride)**	**396.2413**	**54.64**	**2.47**
**para-Toluoyl fentanyl (hydrochloride)**	**398.2358**	**56.39**	**2.55**
**para-chloro Furanyl fentanyl 3-furancarboxamide**	**408.1601**	**55.13**	**2.49**
**N-(2,5-DMA) Fentanyl (hydrochloride)**	**410.2569**	**53.75**	**2.43**

**N-(2C-D) Fentanyl (hydrochloride)**	**410.2569**	**54.18**	**2.45**
**para-Bromofentanyl**	**414.1307**	**51.05**	**2.30**
**Phenoxyacetyl fentanyl (hydrochloride)**	**414.2303**	**59.25**	**2.67**
**N-(2C-E) Fentanyl (hydrochloride)**	**424.2726**	**54.96**	**2.48**
**N-(2C-G) Fentanyl (hydrochloride)**	**424.2726**	**56.17**	**2.54**
**2,3-benzodioxole fentanyl**	**428.2096**	**60.35**	**2.72**
**N-(2C-C) Fentanyl (hydrochloride)**	**430.2023**	**56.68**	**2.56**
**N-(2C-iP) Fentanyl (hydrochloride)**	**438.2882**	**55.13**	**2.49**
**N-(DOET) Fentanyl (hydrochloride)**	**438.2882**	**55.19**	**2.49**
**N-(2C-P) Fentanyl (hydrochloride)**	**438.2882**	**56.23**	**2.54**
**N-(3,4,5-TMA) Fentanyl (hydrochloride)**	**440.2675**	**56.73**	**2.56**
**N-(2C-N) Fentanyl (hydrochloride)**	**441.2264**	**60.96**	**2.75**
**N-(2C-T) Fentanyl**	**442.2290**	**60.08**	**2.71**
**N-(DOC) Fentanyl (hydrochloride)**	**444.2180**	**56.95**	**2.57**
**N-(2C-T-2) Fentanyl (hydrochloride)**	**456.2447**	**60.48**	**2.73**
**N-(2C-TFM) Fentanyl (hydrochloride)**	**464.2287**	**51.24**	**2.31**
**N-(DOBU) Fentanyl (hydrochloride)**	**466.3195**	**57.93**	**2.62**
**N-(2C-T-4) Fentanyl (hydrochloride)**	**470.2603**	**60.26**	**2.72**
**N-(2C-T-7) Fentanyl (hydrochloride)**	**470.2603**	**61.80**	**2.79**
**N-(2C-B) Fentanyl (hydrochloride)**	**474.1518**	**58.45**	**2.64**
**N-(DOB) Fentanyl (hydrochloride)**	**488.1675**	**58.64**	**2.65**
**N-(2C-I) Fentanyl (hydrochloride)**	**522.1379**	**60.55**	**2.73**
**N-(DOI) Fentanyl (hydrochloride)**	**536.1536**	**60.59**	**2.74**

Table S3. Fentanyl analogues that fragment via pathway A, resulting in the abundant formation of product ion A.

**Pathway A**		
Compound	Chemical Formula	Detected Molecular Ion $[M+H]^+$
Norsufentanil	C16H24N2O2	**276.18**
Despropionyl meta-Methylfentanyl	C20H26N2	**294.21**
Despropionyl para-Fluorofentanyl	C19H23FN2	**298.18**
Despropionyl 2'-fluoro ortho-Fluorofentanyl	C19H22F2N2	**316.18**
Acetyl fentanyl	C21H26N2O	**322.20**
2'-methyl Acetyl fentanyl	C22H28N2O	**336.22**
3'-methyl Acetyl fentanyl	C22H28N2O	**336.22**
meta-methyl Acetyl fentanyl	C22H28N2O	**336.22**
(±)-cis-Isofentanyl	C22H28N2O	**336.22**
Fentanyl Methyl Carbamate	C21H26N2O2	**338.20**
2'-methyl Fentanyl	C23H30N2O	**350.24**
3'-methyl Fentanyl	C23H30N2O	**350.24**
4-methyl Fentanyl	C23H30N2O	**350.24**
N,N-Dimethylamido-despropionyl fentanyl	C22H29N3O	**351.23**
meta-fluoro Acrylfentanyl	C22H25FN2O	**352.20**
b-hydroxy fentanyl	C22H28N2O2	**352.22**
2'-Fluorofentanyl	C22H27FN2O	**354.21**
3'-Fluorofentanyl	C22H27FN2O	**354.21**
4'-Fluorofentanyl	C22H27FN2O	**355.22**
meta-methyl Cyclopropyl fentanyl	C24H30N2O	**362.24**
Senecioylfentanyl	C24H30N2O	**362.24**
Tigloyl fentanyl	C24H30N2O	**362.24**
para-methyl Butyryl fentanyl	C24H32N2O	**365.26**
3'-fluoro ortho-Fluorofentanyl	C22H26F2N2O	**372.20**
Furanyl fentanyl	C24H27N2O2	**374.21**
Hexanoyl fentanyl	C25H34N2O	**379.21**
ortho-fluoro Valeryl fentanyl	C24H31FN2O	**382.24**
para-methoxy Methoxyacetyl fentanyl	C23H30N2O3	**383.23**
para-methyl Cyclopentyl fentanyl	C26H34N2O	**391.27**
meta-fluoro Furanyl fentanyl	C24H25FN2O2	**392.19**
Heptanoyl fentanyl	C26H36N2O	**392.28**
Tetrahydrothiophene fentanyl	C24H30N2OS	**395.22**
para-Toluoyl fentanyl	C27H30N2O	**398.24**
2',5'-dimethoxy Fentanyl	C24H32N2O3	396.24
para-chloro Furanyl fentanyl 3-furancarboxamide	C24H25ClN2O2	**409.17**
para-Bromofentanyl	C22H27BrN2O	**414.13**

List Continued

Phenoxyacetyl fentanyl	C27H30N2O2	**415.24**
2,3-Benzodioxole fentanyl	C27H28N2O3	**429.22**
N-(2C-TFM) Fentanyl	C25H31F3N2O3	464.23
N-(2C-I) Fentanyl	C24H31IN2O3	522.14

Table S4. Fentanyl analogs that fragment via pathway B and/or C, thus predominantly generating ions B and/or C.

**Pathway B/C**		
Compound	Chemical Formula	Detected Molecular Ion $[M+H]^+$
N-(Phentermine) Fentanyl	C24H32N2O	364.25
N-(3-ethylindole) Norfentanyl	C24H29N3O	375.23
N-(2-APB) Fentanyl	C25H30N2O2	390.23
N-(6-APB) Fentanyl	C25H30N2O2	390.23
N-(6-APDB) Fentanyl	C25H32N2O2	392.25
N-(MDA) Fentanyl	C24H30N2O3	394.23
2',6'-dimethoxy Fentanyl	C24H32N2O3	396.24
2',4'-dimethoxy Fentanyl	C24H32N2O3	396.24
2',3'-dimethoxy Fentanyl	C24H32N2O3	396.24
3',4'-dimethoxy Fentanyl	C24H32N2O3	396.24
3',5'-dimethoxy Fentanyl	C24H32N2O3	396.24
N-(2,5-DMA) Fentanyl	C25H34N2O3	410.26
N-(2C-D) Fentanyl	C25H34N2O3	410.26
N-(2C-E) Fentanyl	C26H36N2O3	424.27
N-(2C-G) Fentanyl	C26H36N2O3	424.27
N-(2C-C) Fentanyl	C24H31ClN2O3	430.20
N-(2C-iP) Fentanyl	C27H38N2O3	438.29
N-(2C-P) Fentanyl	C27H38N2O3	438.29
N-(DOET) Fentanyl	C27H38N2O3	438.29
N-(3,4,5-TMA) Fentanyl	C26H36N2O4	440.27
N-(2C-N) Fentanyl	C24H31N3O5	441.23
N-(2C-T) Fentanyl	C25H34N2O3S	442.23
N-(DOC) Fentanyl	C25H33ClN2O3	444.22
N-(2C-T-2) Fentanyl	C26H36N2O3S	456.24
N-(DOBU) Fentanyl	C29H42N2O3	466.32
N-(2C-T-4) Fentanyl	C27H38N2O3S	470.26
N-(2C-T-7) Fentanyl	C27H38N2O3S	470.26
N-(2C-B) Fentanyl	C24H31BrN2O3	474.15
N-(DOB) Fentanyl	C25H33BrN2O3	488.17
N-(DOI) Fentanyl	C25H33IN2O3	536.15

Table S5. Fentanyl analogs that fragment via pathway D, thus predominantly fragmenting at the piperidine ring, and resulting in the formation of product ion D.

**Pathway D**		
Compound	Chemical Formula	Detected Molecular Ion $[M+H]^+$
(±)-cis-3-methyl Norfentanyl	C15H22N2O	246.17
Furanyl norfentanyl	C16H18N2O2	270.14
Despropionyl ortho-Fluorofentanyl	C19H23FN2	298.18
2,3-seco-Fentanyl	C22H30N2O	338.24

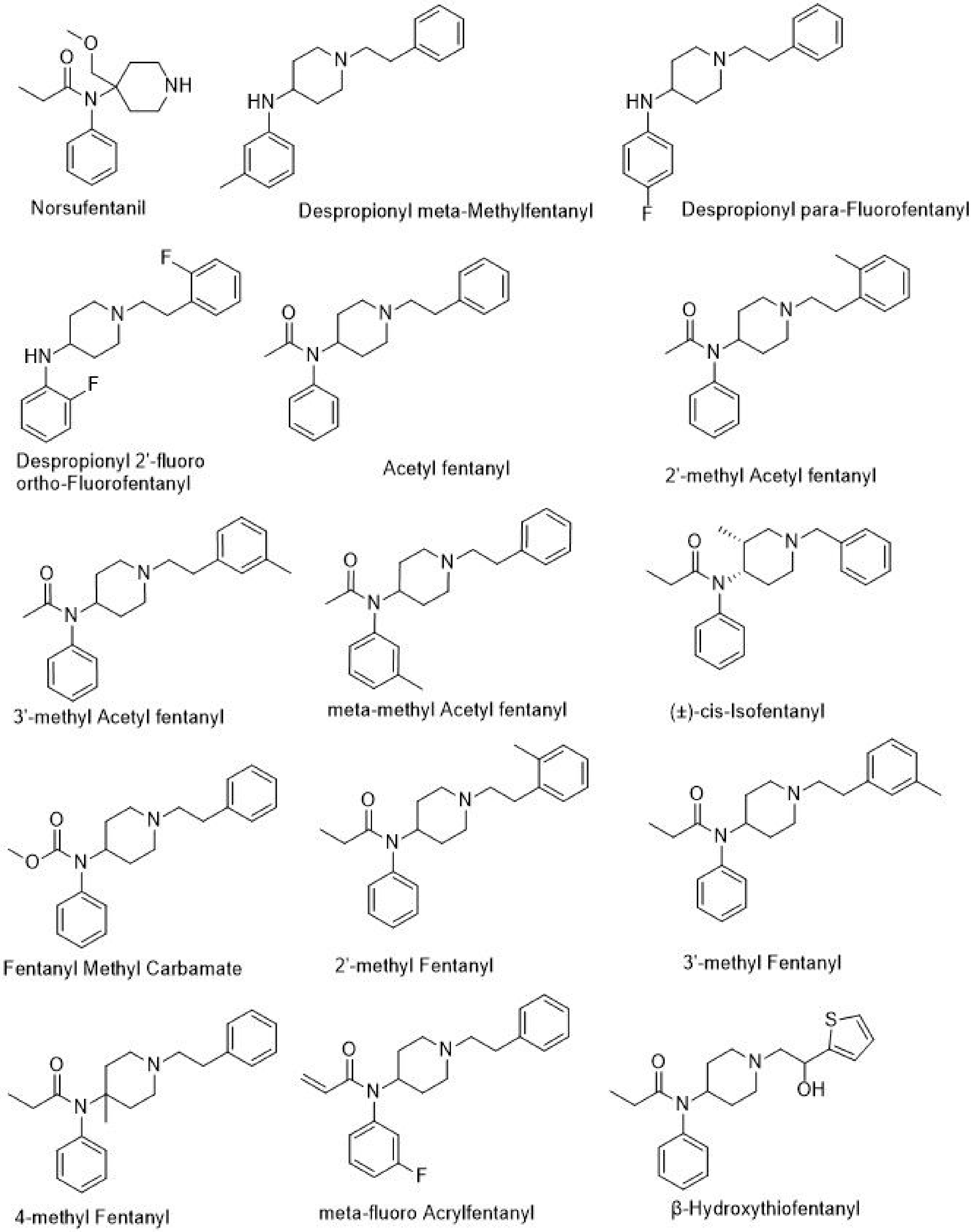

Figure S1. Depiction of 15 analogs that fragment primarily via pathway A.

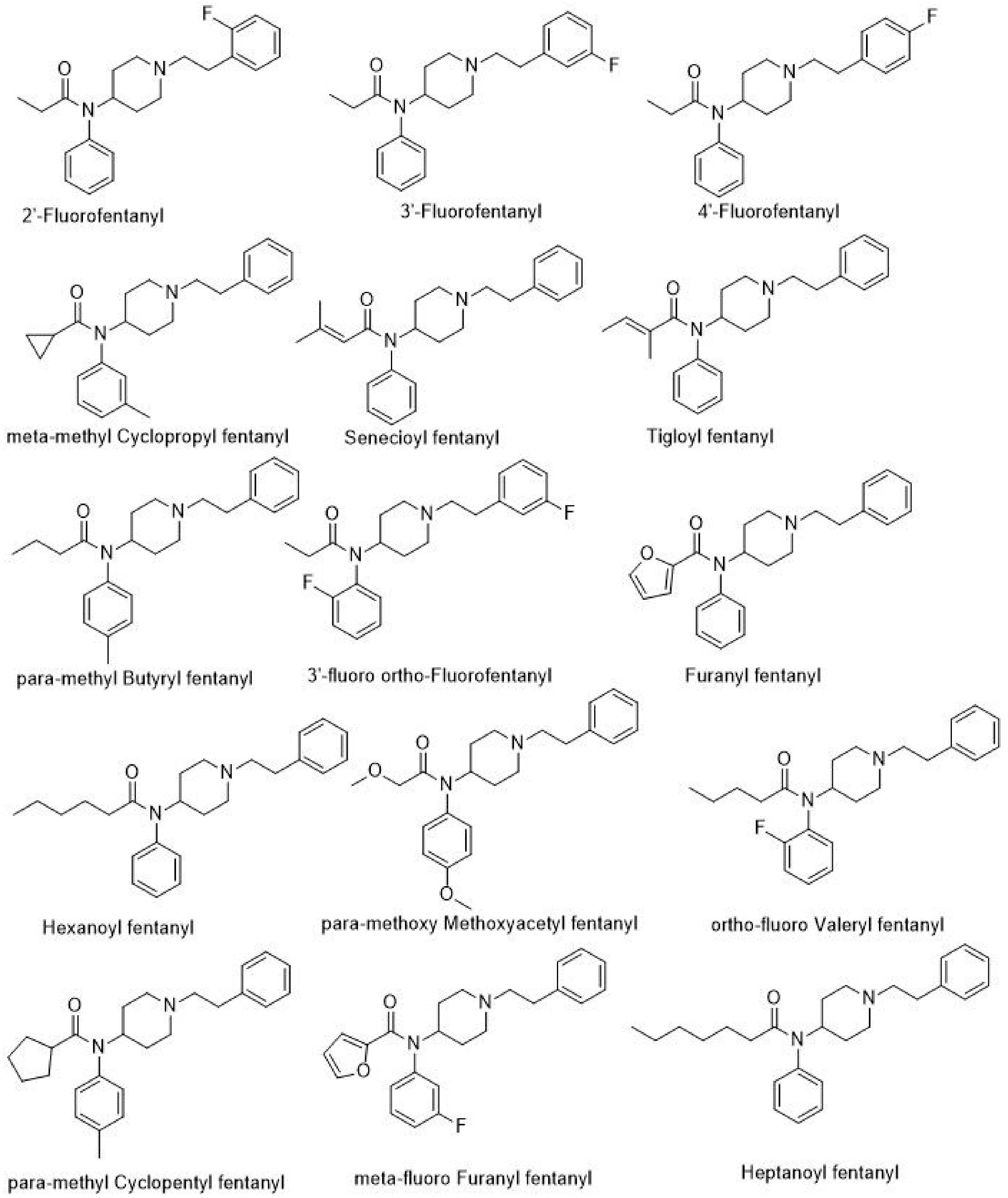

Figure S2. Depiction of another 15 analogs that fragment primarily via pathway A.

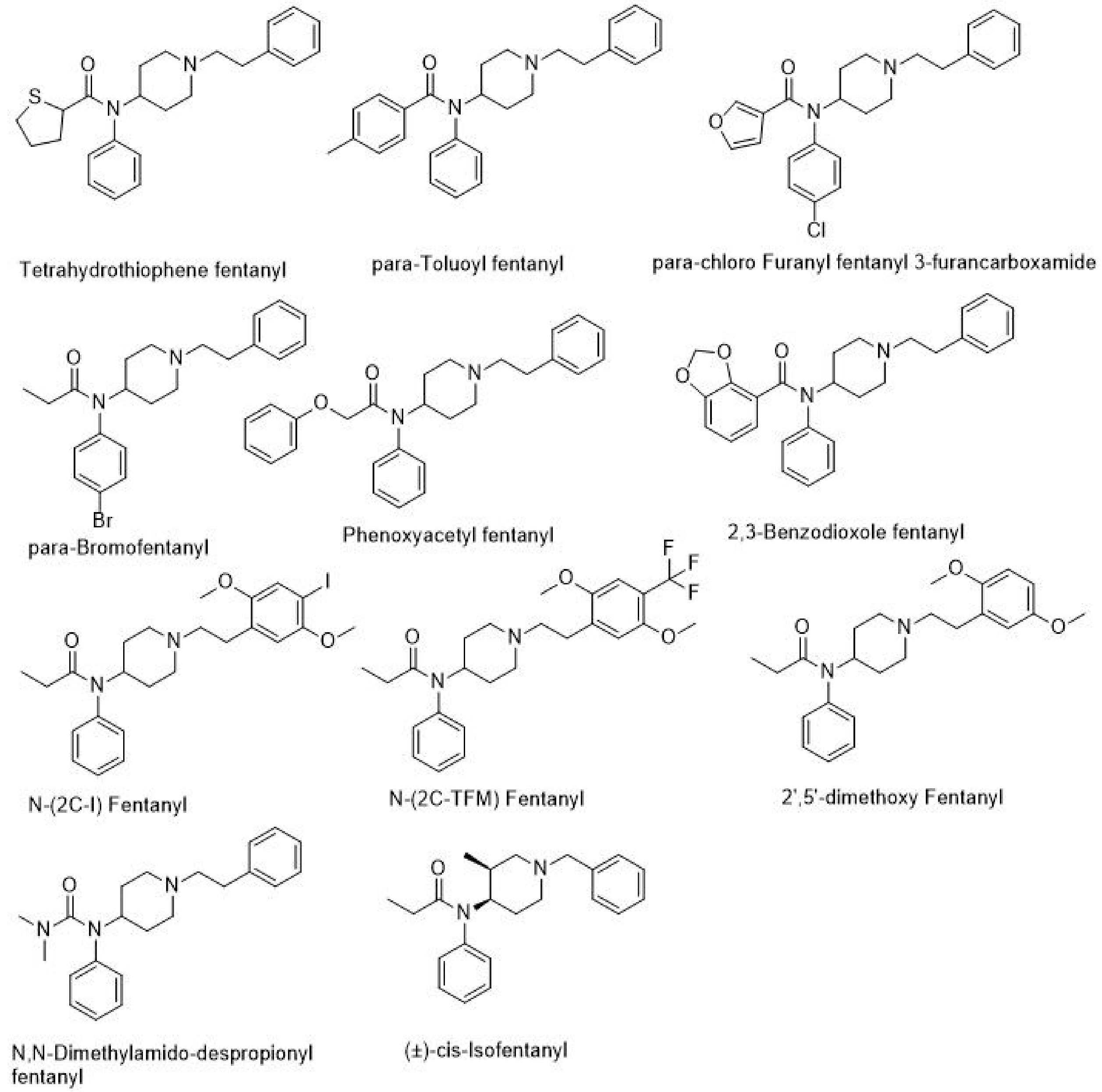

Figure S3. Depiction of the last 10 analogs that fragment primarily via pathway A.

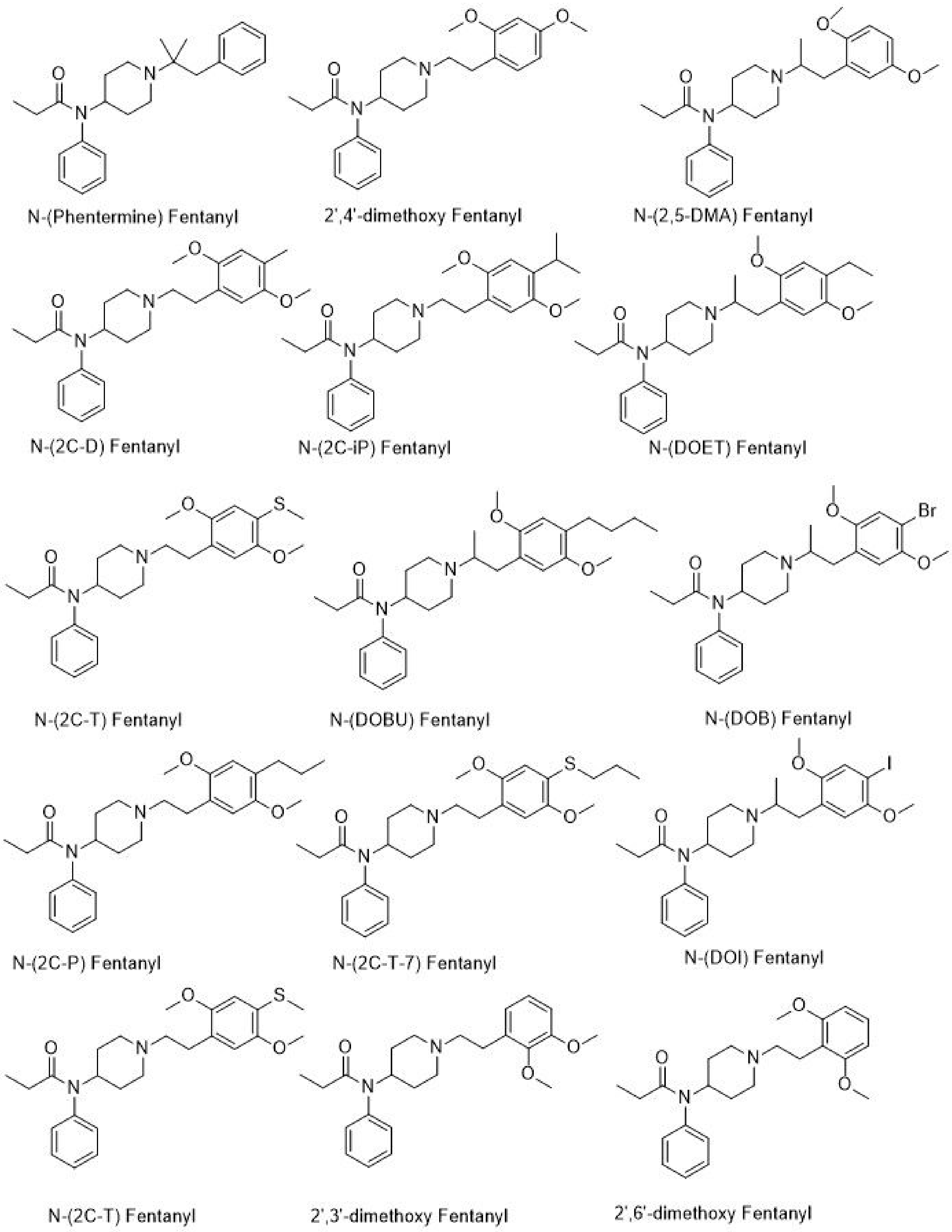

Figure S4. Depiction of 15 analogs that fragment primarily via pathway B/C.

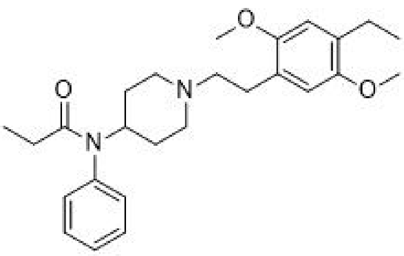

N-(2C-E) Fentanyl

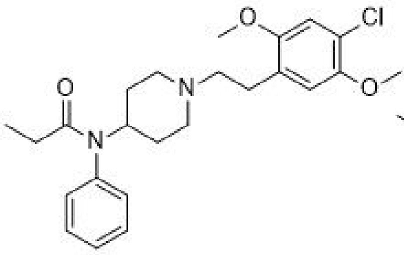

N-(2C-C) Fentanyl

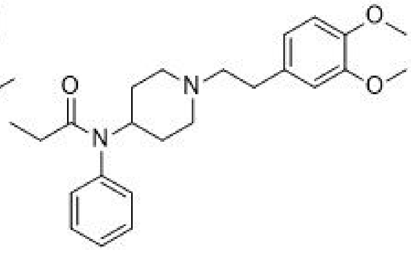

3',4'-dimethoxy Fentanyl

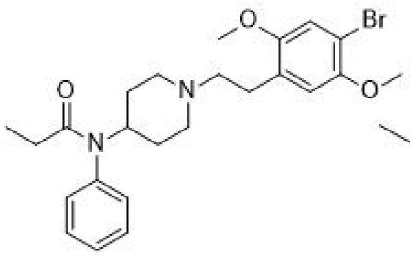

N-(2C-B) Fentanyl

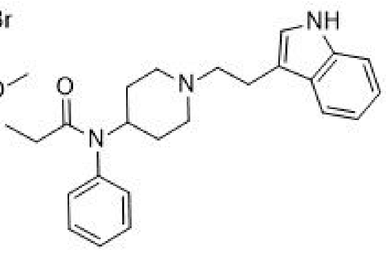

N-(3-ethylindole)

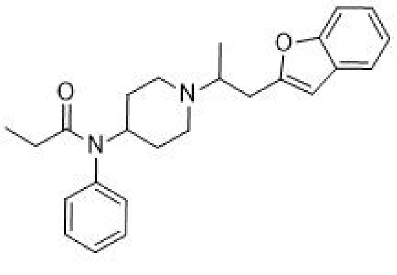

N-(2-APB) Fentanyl

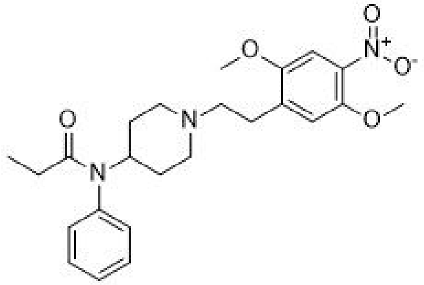

N-(2C-N) Fentanyl

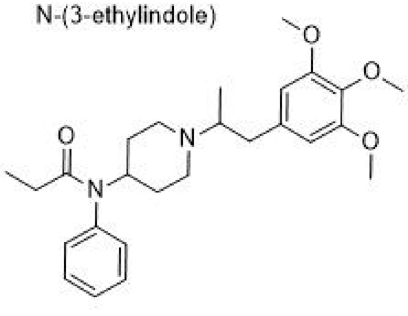

N-(3,4,5-TMA) Fentanyl

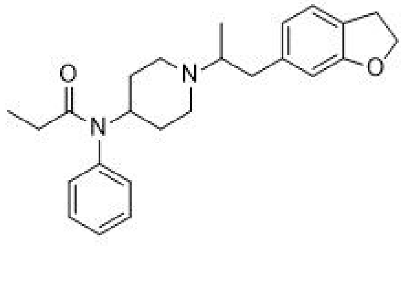

N-(6-APDB) Fentanyl

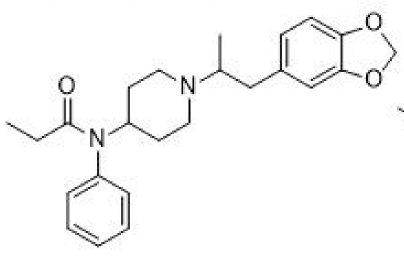

N-(MDA) Fentanyl

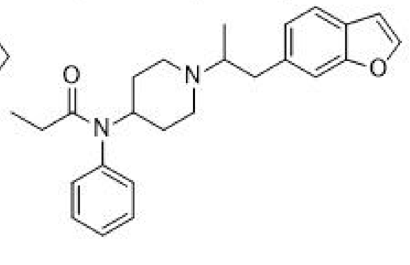

N-(6-APB) Fentanyl

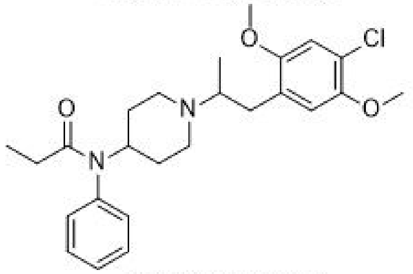

N-(DOC) Fentanyl

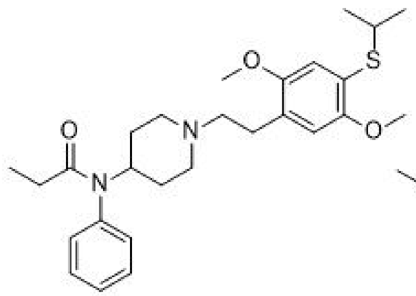

N-(2C-T-4) Fentanyl

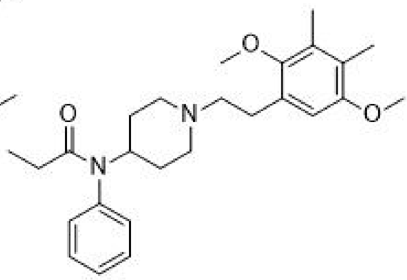

N-(2C-G) Fentanyl

Figure S5. Depiction of the last 14 analogs that fragment primarily via pathway B/C.

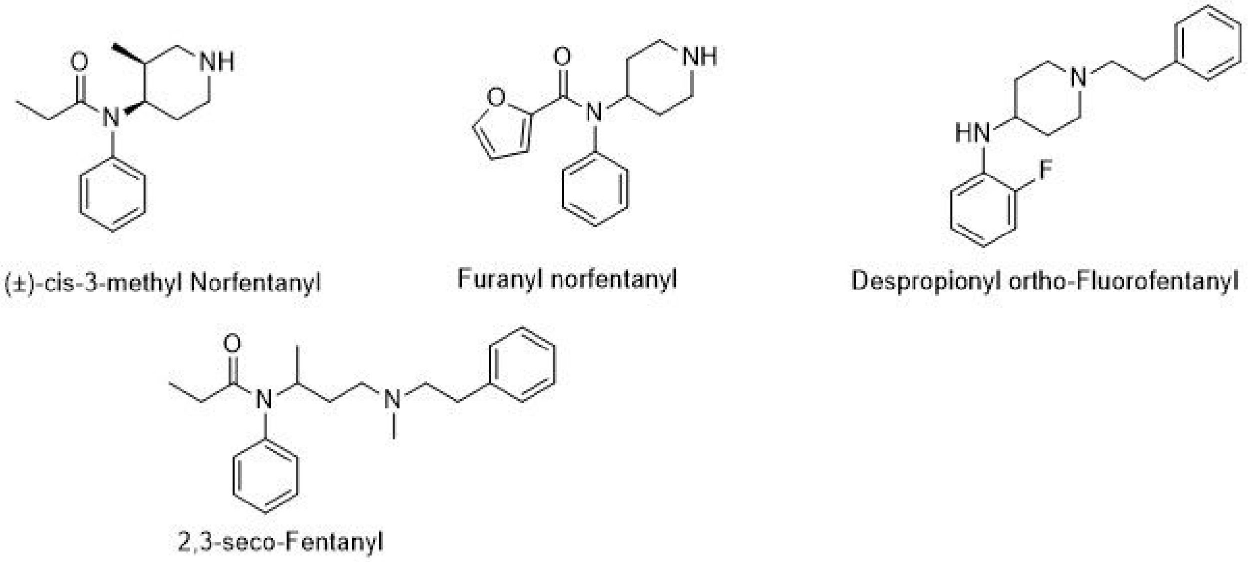

Figure S6. Depiction of 4 analogs that follow primarily pathway D.

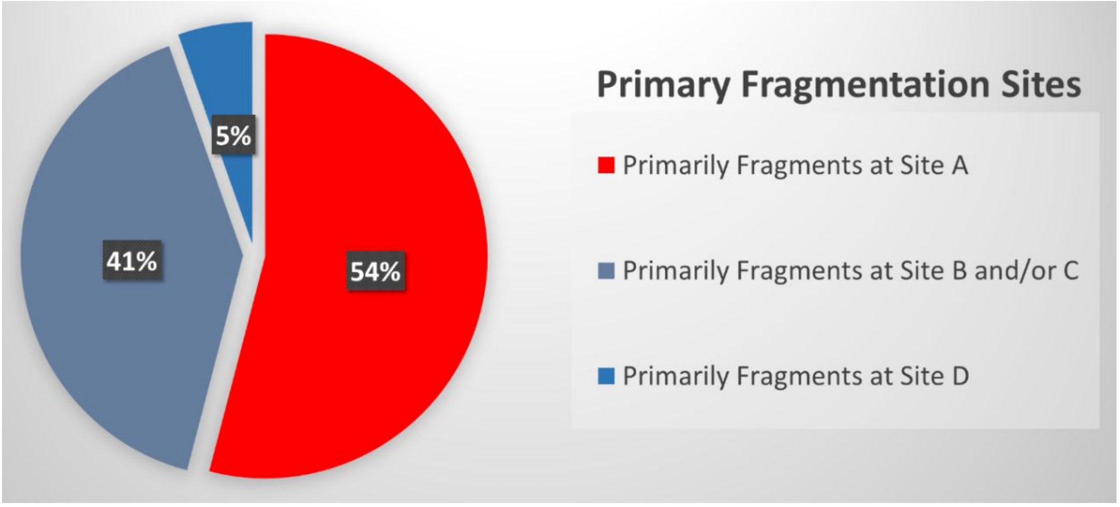

Figure S7. Pie chart depicting the distribution of primary fragmentation sites among the 74 fentanyl analogs analyzed. Of the fentanyl analogs studied, 54% primarily fragmented at site A (the amide N-C4 bond), while 40% fragmented at sites B and/or C (the n-alkyl chain). Only 5% exhibited fragmentation primarily at site D (the piperidine ring).

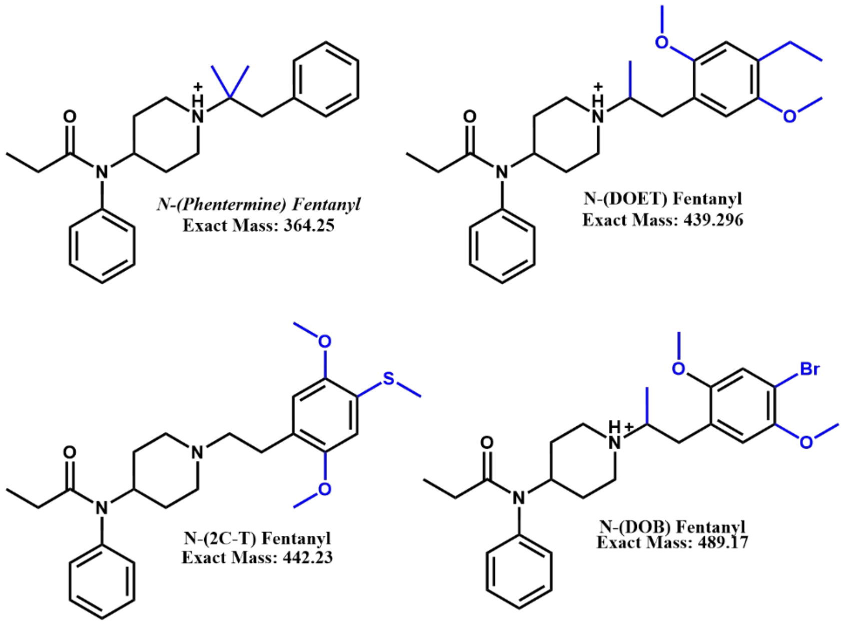

Figure S8. Four of the eleven analogs that produce solely product ion B at 10-50 V. These analogs generally contain a methyl at the alpha carbon of the alkyl chain and three functional groups at the phenyl moiety, two of which are methoxy groups.

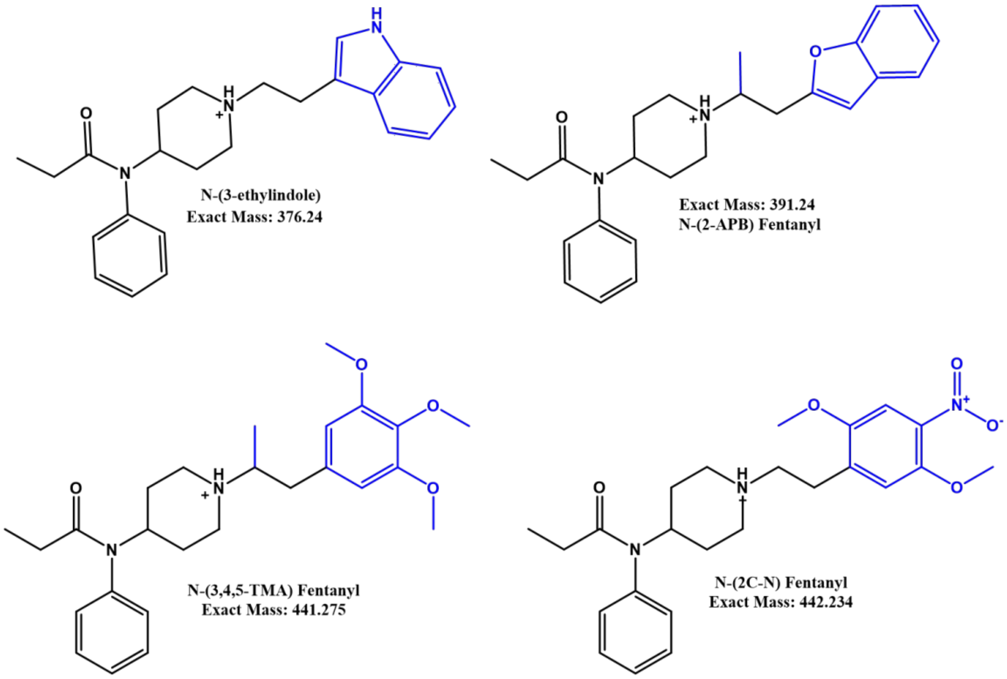

Figure S9. Four of the ten fentanyl analogs that produced product ion C at 20- 50V. These specific compounds contain highly stabilized and substituted R4 groups that drive fragmentation to occur primarily at site C.

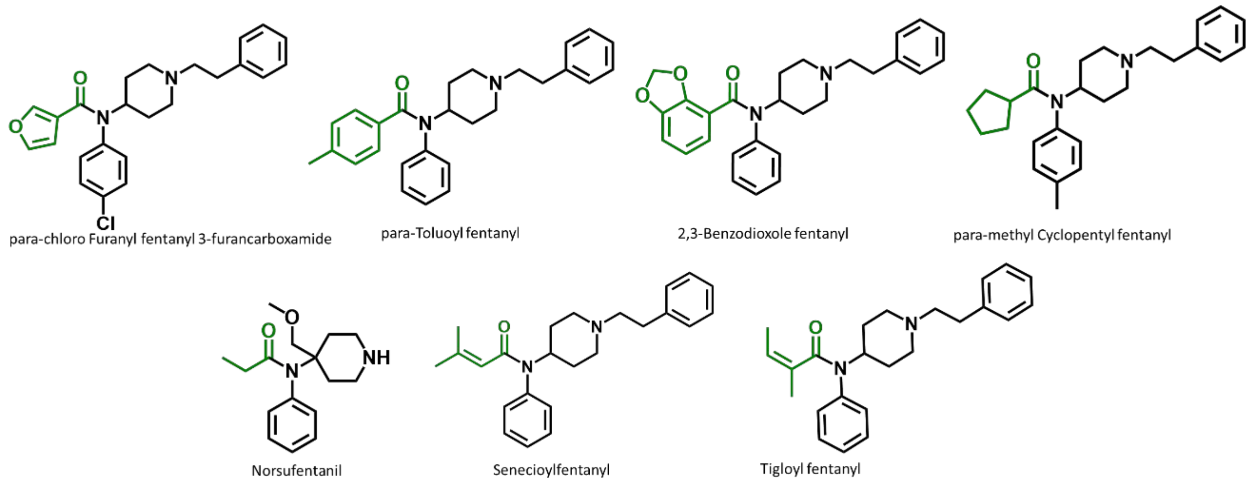

Figure S10. Illustration of pathway E depicting the structures of norsufentanil, senecioylfentanyl, tigloylfentanyl, para-toluoylfentanyl, para-chloro-furanylfentanyl 3-furancarboxamide, 2,3-benzodioxolefentanyl, and para-methyl cyclopentyl fentanyl with corresponding functional group R1 highlighted in green.

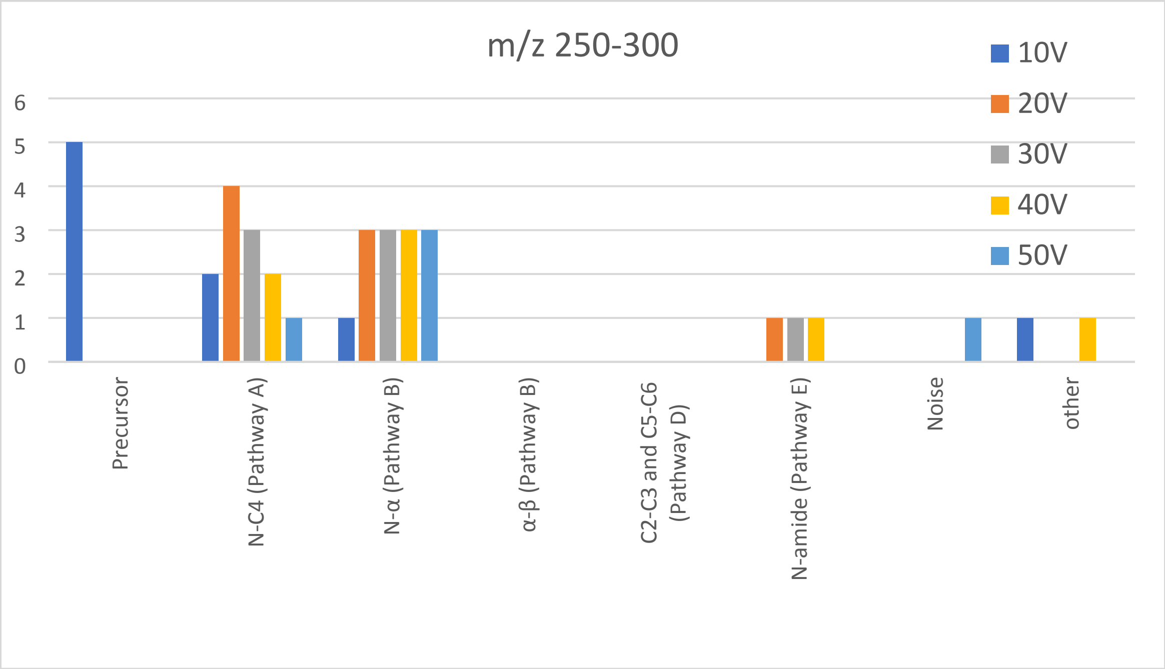

Figure S11. Fragmentation data at different voltages for the 250-300 m/z compounds.

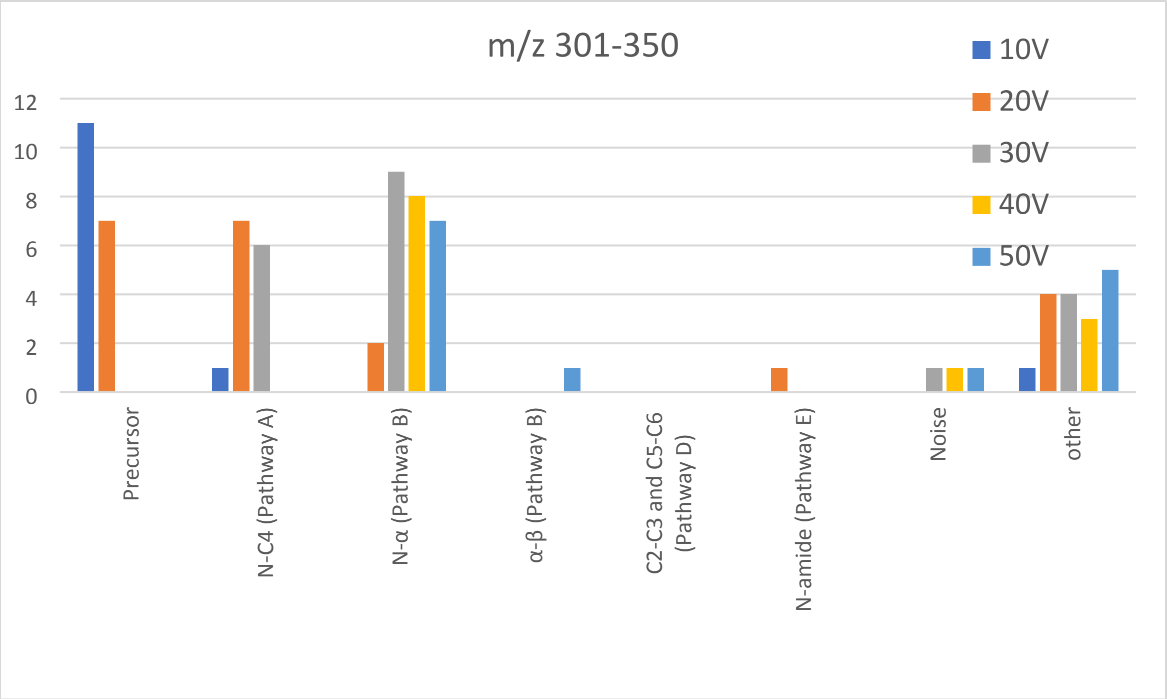

Figure S12. Fragmentation data at different voltages for the 301-350 m/z compounds.

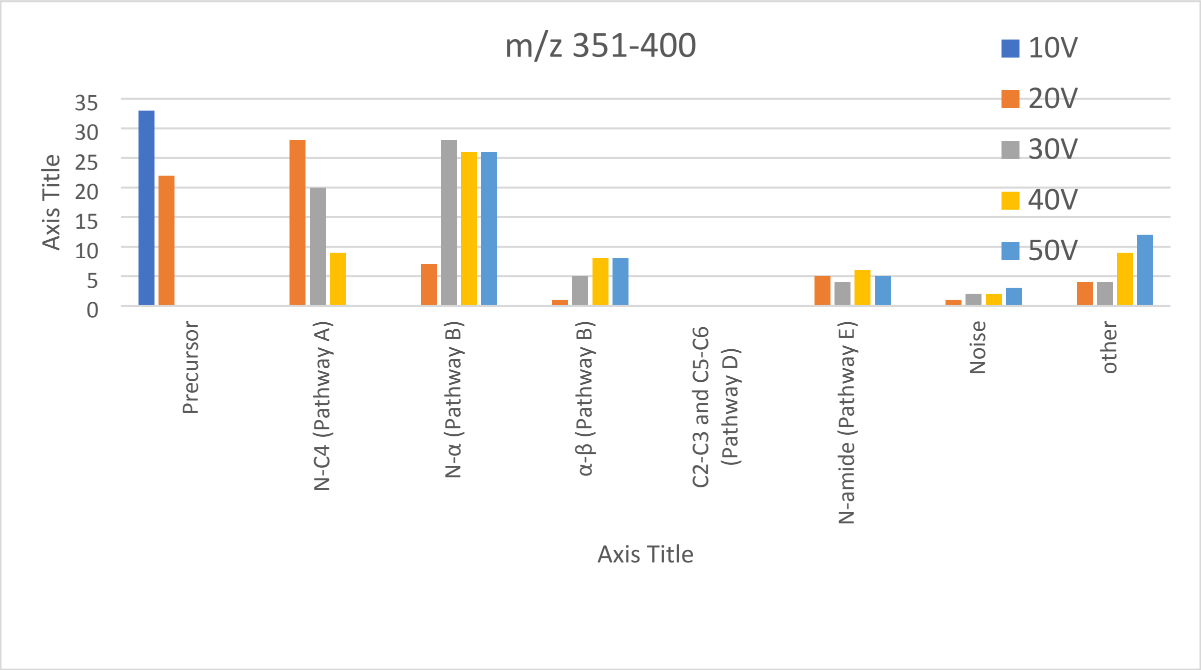

Figure S13. Fragmentation data at different voltages for the 351-400 m/z compounds.

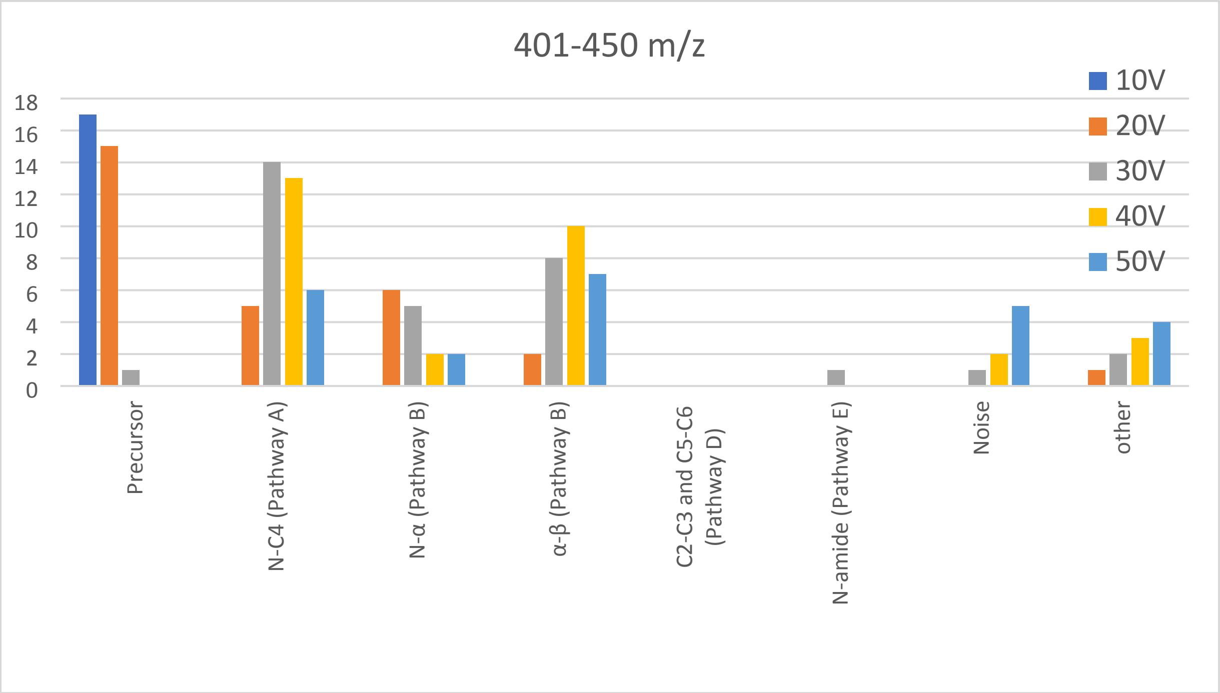

Figure S14. Fragmentation data at different voltages for the 401-450 m/z compounds.

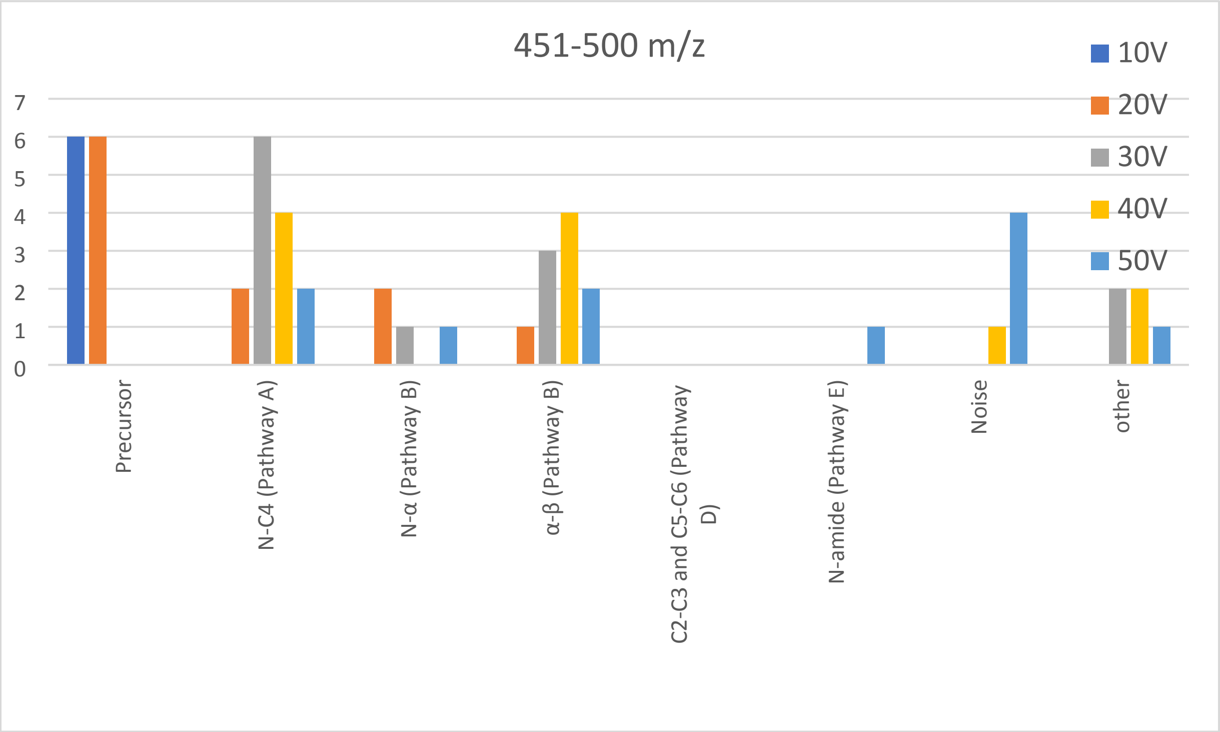

Figure S15. Fragmentation data at different voltages for the 451-500 m/z compounds.

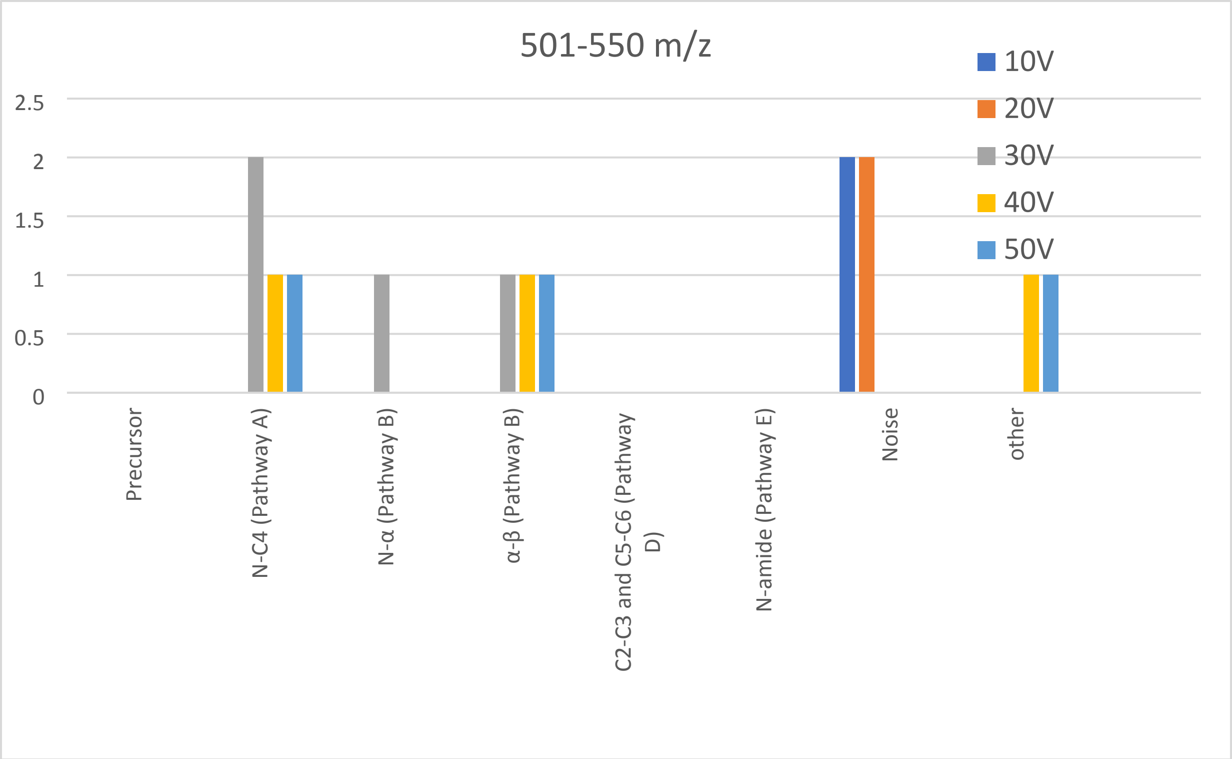

Figure S16. Fragmentation data at different voltages for the 501-550 m/z compounds.

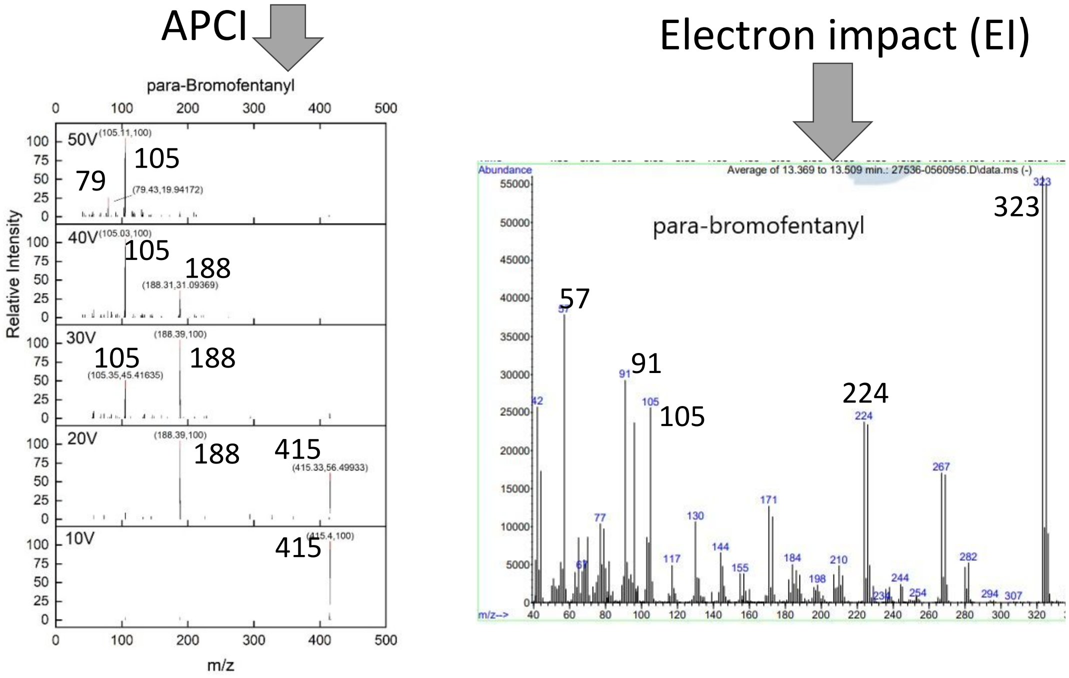

Figure S17. Illustration of APCI data (left) from our study and EI data (right) for para-bromofentanyl. (EI data used with permission from Cayman Chemical).
